# Development of a Scalable Synthetic Route to (1*R*,5*R*)-2,2-Dimethoxybicyclo[3.1.0]hexan-3-one: An Important Intermediate in the Synthesis of Lenacapavir

**DOI:** 10.1021/acs.oprd.4c00527

**Published:** 2025-02-26

**Authors:** Aline Nunes De Souza, Nagaraju Sakkani, Daryl Guthrie, Rajkumar Lalji Sahani, John M. Saathoff, Samuel R. Hochstetler, Justina M. Burns, Saeed Ahmad, G. Michael Laidlaw, B. Frank Gupton, Douglas A. Klumpp, Limei Jin

**Affiliations:** Medicines for All Institute, Virginia Commonwealth University, Richmond, Virginia, 23284-3068, United States

**Keywords:** asymmetric synthesis, (*R*)-epichlorohydrin, telescoping, I_2_-promoted hydroxylation, Albright–Goldman oxidation, process development, lenacapavir

## Abstract

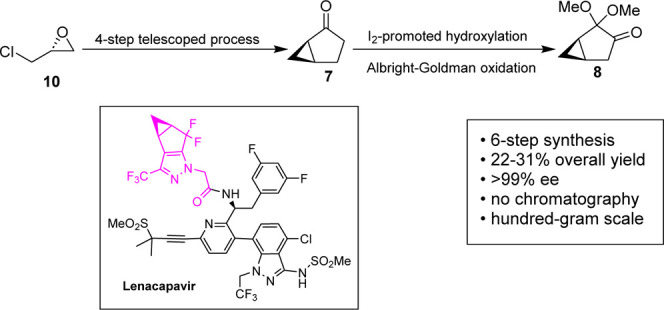

(1*R*,5*R*)-2,2-Dimethoxybicyclo[3.1.0]hexan-3-one is used in the asymmetric synthesis of lenacapavir. Herein, we report an enantioselective synthesis of this important chiral intermediate from the inexpensive commodity (*R*)-epichlorohydrin. This synthetic method comprises 6 steps, including a 4-step telescoped bicyclic ketone synthesis, I_2_-promoted hydroxylation, and an Albright–Goldman oxidation. This sequence affords (1*R*,5*R*)-2,2-dimethoxybicyclo[3.1.0]hexan-3-one in an overall 25% isolated yield as an enantiomerically pure compound. The entire process has been successfully demonstrated on a hundred-gram scale.

## Introduction

The human immunodeficiency virus (HIV), and its progression to acquired immunodeficiency syndrome (AIDS), constitutes one of the most serious health threats in the world. There are currently more than 1 million new cases per year, and a total of 40 million people globally are infected with HIV.^1^ This includes more than 1.5 million children. There are annually over 600,000 deaths worldwide from AIDS and more than 40 million deaths since the start of the epidemic.^2^

Recently, a promising new therapy was discovered for the treatment of HIV. Lenacapavir (trade named Sunlenca) is a first-in-class drug that targets the HIV capsid protein, disrupting the function of this protein across multiple steps in the viral life cycle.^3−5^ This leads to stunning levels of biological activity, including activity against multidrug-resistant strains and all subtypes of HIV-1. This activity has been demonstrated at picomolar concentrations.^6^ Top-line results from Phase 3 clinical trials, reported in June 2024, showed that lenacapavir was safe and 100% effective as a long-acting HIV pre-exposure prophylaxis (PrEP) among cisgender women.^7^ As such, lenacapavir is likely to become a vital tool in the treatment of HIV infections; therefore, there is a need for an improved and low-cost synthesis.^8^

**Figure 1:**
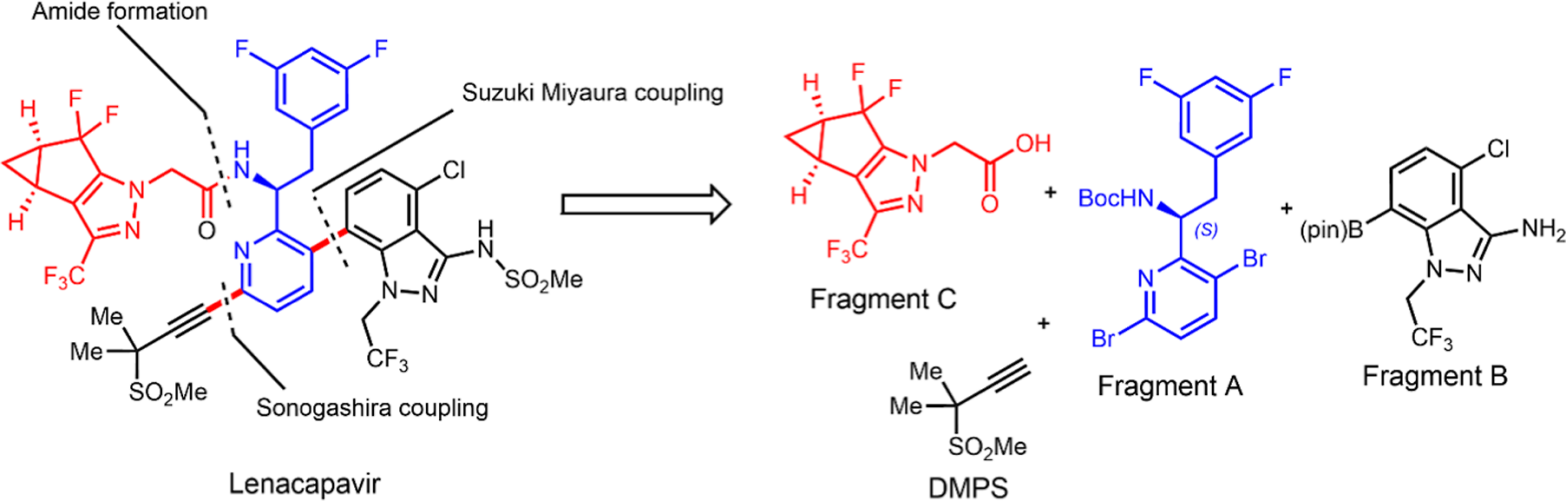
Retrosynthetic disconnection of lenacapavir into synthetic fragments.

**Scheme 1:**
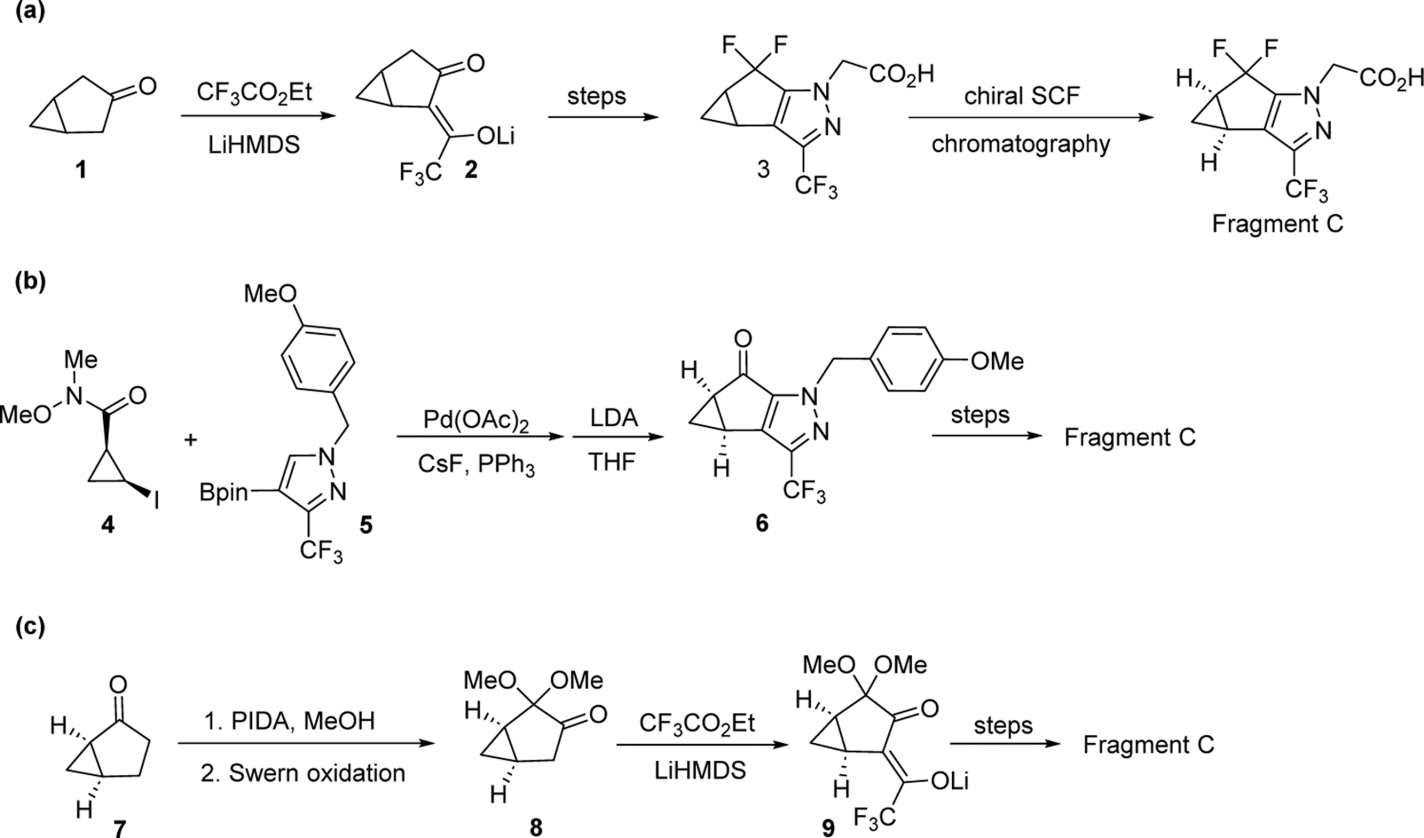
Synthetic Approaches (a–c) to Obtain Fragment C as a Single Enantiomer Disclosed by Gilead

Lenacapavir was first reported by Gilead Sciences in a family of patents and publications in 2018–2020.^9−12^ The Gilead synthesis of lenacapavir involves the coupling of three advanced intermediates (or fragments) and an alkynyl sulfone (DMPS, Figure 1). Lenacapavir features two fixed stereocenters and has the potential for axial chirality of its biaryl bond. Fragment A contains a Boc-protected chiral amine.^13−15^ Fragment B is a boronic ester of an 1*N*-trifluoroethyl-3-amino-indazole compound. We recently reported efficient, cost-effective synthetic routes to each of these fragments, A^13^ and B.^16^ Fragment C’s chirality originates in the cyclopropane ring junction, which leads to synthetic challenges in producing the single enantiomer required in the final active pharmaceutical ingredient. Gilead’s original disclosures described two approaches to Fragment C, a racemic synthesis followed by resolution of the desired enantiomer and single enantiomer synthesis (Scheme 1). As shown in Scheme 1a, the resolution approach started from the synthesis of a racemic mixture of Fragment C, which was obtained through a Claisen condensation of bicyclic ketone **1** followed by a Knorr cyclization. The desired enantiomer of Fragment C is then isolated using supercritical fluid chromatography with a chiral stationary phase.^9^ Although this provides the required single enantiomer, the undesired enantiomer cannot be recycled; at least 50% of the crude product is effectively lost. Moving the enantiomer isolation earlier in the process to the cyclopropane intermediate also resulted in a 50% yield loss.^10^

To prepare Fragment C as a single enantiomer, two methods were developed (Scheme 1b,c). Chiral cyclopropane (**4**) was used to make the single enantiomer ketone (**6**) in a two-step sequence invoking a Suzuki–Miyaura coupling followed by intramolecular arylation of the Weinreb amide.^10^ While compound **6** may be converted to Fragment C, the method involves an expensive cyclopropane intermediate and a Pd catalyst (Scheme 1b). In another approach, (1*R*,5*S*)-bicyclo[3.1.0]hexan-2-one (**7**) provided access to the desired enantiomer for Fragment C (Scheme 1c).^10,17^ With oxidation of **7** to the enantiomerically pure acetal (**8**), the Claisen condensation intermediate (**9**) leads into pyrazole formation and fluorination steps.^10^ These approaches gave Fragment C as a pure enantiomer—without incurring the costly removal of the undesired enantiomer.^18−20^

**Scheme 2:**
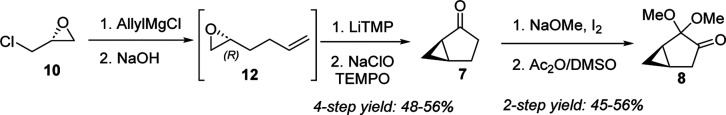
Asymmetric Synthesis of (1*R*,5*R*)-2,2-Dimethoxybicyclo[3.1.0]hexan-3-one (**8**) from (*R*)-Epichlorohydrin (**10**)

Herein, we describe our work in the development of a scalable synthetic route to (1*R*,5*R*)-2,2-dimethoxybicyclo[3.1.0]hexan-3-one (**8**), an intermediate in the synthesis of Fragment C of lenacapavir. Our approach leverages a 4-step telescoped process to synthesize (1*R*,5*S*)-bicyclo[3.1.0]hexan-2-one (**7**) from readily available (*R*)-epichlorohydrin (**10**). Using an I_2_-promoted hydroxylation and Albright–Goldman oxidation to further transform compound **7**, the optimized steps provide (1*R*,5*R*)-2,2-dimethoxybicyclo[3.1.0]hexan-3-one (**8**) in 25% overall yield, from a 6-step synthetic sequence (Scheme 2).

## Results and Discussion

**Scheme 3:**
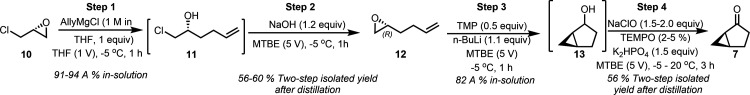
Step-Wise Synthesis of (1*R*,5*S*)-Bicyclo[3.1.0]hexan-2-one (**7**)

Unlike the conditions reported by Hodgson et al.,^21^ we recently reported a 2-step process (ring-opening and ring closure) to synthesize (*R*)-(+)-1,2-epoxy-5-hexene (**12**) from readily available (*R*)-epichlorohydrin (**10**) without the use of CuI as catalyst.^22^ The ring-opening reaction of (*R*)-epichlorohydrin (**10**) with allylmagnesium chloride (allylMgCl) afforded crude chlorohydrin **11** in 91–94 A % (GCMS-TIC). Sodium hydroxide-mediated ring closure of **11** produced **12** in 80–90% in-solution yield. The isolated yield of **12** from **10** was 55–60% with a purity of 98–99 A % (GCMS-TIC) after distillation. Chiral epoxide **12** was advanced to the bicyclic ketone **7** via Hodgson cyclopropanation then oxidation of the incipient cycloproylcarbinyl alcohol (Scheme 3).^17^

As depicted in Scheme 3, treatment of **12** with lithium 2,2,6,6-tetramethylpiperidide [formed in situ from *n*-BuLi and 2,2,6,6-tetramethylpiperidine (TMP)] enabled the formation of crude **13** in 82 A % (GCMS-TIC). TEMPO-bleach oxidation^23^ of crude **13** delivered the bicyclic ketone **7**. Pure ketone **7** was obtained after distillation and the isolated yield of this two-step process (from **12**) was 56% with a purity of 93 A % (GCMS-TIC). The overall isolated yield of **7** from **10** (4 steps in total) was low, only 31–33%, mainly due to a significant amount of product loss in each distillation. To improve the isolated yield of **7**, our efforts were then focused on telescoping the process development and synthesis of **7**, with the aim of minimizing the number of distillations of the intermediates. The telescoped process development for the ring-opening reaction of **10** and ring-closure of **11** to synthesize **12** was disclosed in our recent publication.^22^

The high purity profile of crude epoxide **12** provides possible telescoping options for the subsequent Hodgson cyclopropanation. As shown in Table 1, a hundred-gram scale reaction of (*R*)-epichlorohydrin (**10**) with allylMgCl afforded an MTBE solution of **11**, which was then treated with NaOH (2 M), affording a MTBE solution of **12** with a 98–99 A % (GCMS-TIC) after an aqueous workup. Karl Fischer (KF) titration of the resulting solution afforded a moisture content of ∼1.5%. As the subsequent Hodgson reaction is moisture sensitive,^24^ it was critical to dry the reaction mixture of **12** before advancing to **13**. Azeotropic distillation enabled access to MTBE solutions of **12** with less than 0.2 wt % moisture by KF titration (details in the Supporting Information).^25^ Under the reported protocol for the Hodgson reaction,^17^ its performance using the solution of **12** was the same as had been observed using pure **12**. As shown in Table 1, treatment of **12** (in MTBE) with 1.1 equiv of *n*-BuLi in the presence of 0.5 equiv of TMP at 0 °C afforded **13** in 85–91 A % (GCMS-TIC). After an aqueous workup, the resulting mixture of **13** in MTBE was concentrated to about 5 volumes and then submitted to the oxidation step. Oxidation of crude **13** with NaClO/TEMPO successfully afforded **7** in 87–98 A %. As summarized in Table 1, this 4-step telescoped protocol featured a high overall isolated yield of **7** (48–55% vs 31–33% in the stepwise protocol), minimal number of distillations (only one distillation of a solution of **7** in the 4-step telescoped protocol), and good reproducibility and scalability (demonstration on hundred-gram scale) (Table 1, entries 1–3).

**Table 1:**
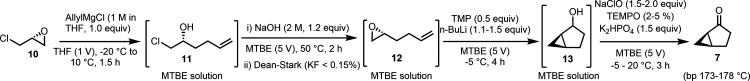
Scale-Up Summary of Four-Step Telescoped Synthesis of (1*R,*5*S*)-Bicyclo[3.1.0]hexan-2-one (**7**) from (*R*)-Epichlorohydrin (**10**)

		A % by GCMS-TIC^a^				7 (after distillation)^b^				
entry	**10** (g)	**11**^c^	**12**^d^	**13**^e^	**7**^f^	amount (g)	purity^g^ (wt %)	purity^h^ (A %)	4-step yield^i^ (%)	ee (%)^j^
1	148	95	99	91	98	76	81	97^k^	48	100
2	148	95	99	89	87	91	94	92^l^	55	100
3	137	93	98	85	88	88	83	98^m^	53	100

a All data was obtained from crude reaction mixture of each step after aqueous workup; area % (A %) was measured by GC–MS total ion chromatogram.

b Distillation conditions: MTBE was removed under 146 mbar at 40 °C and **7** was distilled under 2 mbar at 40 °C.

c The resulting reaction mixture of **10** and allylMgCl was quenched by MeOH, acidified with aq HCl (2 M), and extracted with MTBE. The solution of **11** in MTBE was washed with aq HCl (2 M) and water then used for the next step.

d The resulting solution of **12** in MTBE (KF): ∼1.5%) was heated at 70 °C to remove water by azeotropic distillation to achieve a KF of 0.15% for the next step.

e The resulting solution of **13** in MTBE was concentrated to ∼800 mL under 200 mbar at 40 °C for the next step; containing ∼3–4 A % of epichlorohydrin, ∼5 A % of **12**.

f The resulting solution of **7** in MTBE was concentrated under 200 mbar at 40 °C to remove most of the MTBE for further distillation to afford **7**.

g Wt % was measured by GCMS.

h A % was measured by GCMS-TIC.

i Corrected isolated yield.

j Enantiomeric excess (ee) was measured by chiral GC.

k Containing unknown peaks (<1 A % each).

l Containing TEMPO (4 A %), **13** (2 A %) and several unknown peaks (<1 A % each).

m Containing **13** (1.7 A %).

Oxidation of **7** with phenyliodine(III) diacetate (PIDA) was performed according to the literature.^10^ Reaction of **7** and PIDA in the presence of KOH afforded **14** in 54–71 A % (GCMS-TIC) (Table 2, entries 1–2). Iodobenzene was observed as a byproduct, requiring column chromatography for purification. The high cost and low market availability of PIDA made PIDA-promoted hydroxylation challenging to scale up. To ensure a more practical and affordable process to access **14**, we aimed to (1) not only utilize an inexpensive oxidant in this hydroxylation but also (2) eliminate the need for column purification. At the outset of our studies, we evaluated a variety of readily available oxidants in the hydroxylation of **7** (Table 2, entries 3–6). However, no hydroxylation occurred when employing TCCA/TEMPO,^26^ I_2_/DMSO, or NBS/DMSO^27^ as the oxidative systems (Table 2, entries 3–4). OXONE afforded 15 A % (GCMS-TIC) of α-hydroxylated product,^28^ but attempts to further improve the yield failed; an increase of equivalents of OXONE, elongation of reaction time, or enhancement of reaction temperature did not improve the yield (Table 2, entry 5).

**Table 2:**
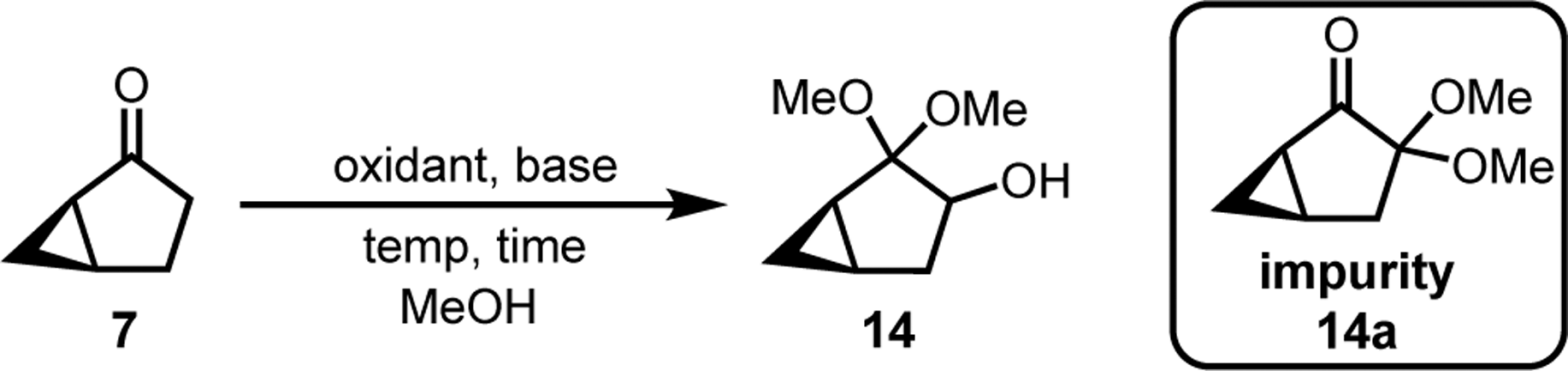
Conditions Screen of Hydroxylation of Ketone **7** to Prepare Alcohol **14**

entry^a^	variations of the baseline conditions	14 (A %, GCMS-TIC)
1	baseline conditions^b^: PhI(OAc)_2_ (1.5 equiv), KOH (3.5 equiv), MeOH (13 V), 0–25 °C, 15 h	71^c^
2	PhI(OAc)_2_ (1.0 equiv), KOH (3.5 equiv), MeOH (13 V), 0–25 °C, 15 h	54
3	TCCA (1.05 equiv)/TEMPO (0.01 equiv), DCM (16 V), 0–25 °C, 16 h	ND
4	I_2_ (0.2 equiv) or NBS (0.2 eq), DMSO, 60 °C, 24 h	ND
5	OXONE (monopersulfate, 2.7 equiv), TFAA (7 equiv), PhI (0.2 equiv), H_2_O (42 V)/CH_3_CN (125 V), 40–90 °C, 22 h	15^d^
6^e^	I_2_ (1.2 equiv), KOH (2.4 equiv), MeOH (37 V), 0–25 °C, 14 h	61
7	I_2_ (1.2 equiv), NaOMe (2.4 equiv), MeOH (37 V), 0–25 °C, 14 h	80
8	I_2_ (1.2 equiv), NaOMe (2.4 equiv), MeOH (37 V), –9–0 °C, 12 h	86
9	I_2_ (1.2 equiv), NaOMe (2.4 equiv), MeOH (37 V), –9–0 °C, 12 h	87^f^

a In these condition screens, racemic **7** was used.

b Typical procedure with PIDA unless otherwise stated: KOH (3.5 equiv) dissolved in MeOH (13 V) at 55 °C, cooled to 0–6 °C, **7** (1 g) was added slowly, stirred at 0–6 °C for 45 min, PIDA (1.5 equiv) was added slowly at 0–6 °C and stirred at the same temperature until completion (∼15 h), purified by column chromatography (SiO_2_, hexanes/EtOAc, 8/2, v/v).

c Isolated yield: 61% after column chromatography.

d 15 A % (GCMS-TIC) of the α-hydroxylated product was formed, 85 A % of **7** remained, an increase of equivalents of OXONE, elongation of reaction time, or enhancement of reaction temperature resulted in no improvement.

e Typical procedure with I_2_ unless otherwise stated: NaOMe (2.4 equiv) in MeOH (19 V) was cooled to 0–6 °C, **7** (1 g) was added slowly, 10 min later, I_2_ (1.2 equiv) in MeOH (18 V) was added slowly at 0–6 °C, the mixture was stirred until completion (∼12–14 h). The reaction mixture was concentrated to remove MeOH under reduced pressure at rt. The residue was extracted with DCM, and washed with sat. Na_2_SO_3_. All DCM layers were concentrated and distilled at 60–70 °C under vacuum (10 Pa) to afford **14**, 5–7 A % (GCMS-TIC) **14a** (tentative) was constantly formed.

f 30 g of **7**, isolated yield: 73% after distillation.

I_2_ with KOH delivered **14** in moderate yield (61 A %, GCMS-TIC) (Table 2, entry 6).^29^ I_2_ proved superior to PIDA; the former did not form iodobenzene as a byproduct, and it is more readily available. Streamlining the purification eliminated the need for column chromatography. Furthermore, NaOMe delivered better results than the incumbent base, KOH. Treatment of **7** and NaOMe with a solution of I_2_ in MeOH at 0–25 °C afforded **14** in 80 A % (GCMS-TIC) (Table 2, entry 7). Notably, the slow addition of I_2_ solution in MeOH was imperative to achieve a high yield. Lowering the reaction temperature from −9 to 0 °C further improved the A % of **14** to 86 A % (GCMS-TIC) (Table 2, entry 8). The optimal conditions for this oxidation were demonstrated on a 30 g scale, providing **14** in 87 A % (GCMS-TIC) in crude and 75% isolated yield after distillation (Table 2, entry 9).^30^

Despite improvements in yield and reaction purity, opportunities remained to address sustainability issues. A total of 37 V of solvents were consumed under the aforementioned hydroxylation conditions. An investigation to lower the solvent volume was conducted. Ultimately, reaction of NaOMe (6 V), solid I_2_, and **7** in order afforded **14** in excellent yield and this hydroxylation process dramatically reduced the total solvent volume to 6 V, which is suitable for further scale-up (details in the Supporting Information).

With a practical method for the preparation of **14** in hand, our focus was then shifted to the next oxidation of **14** to furnish desired ketone **8**. Swern oxidation of **14** accomplished the formation of **8** efficiently (Table 3, entry 1).^31^ However, a cryogenic condition was needed in this transformation (Table 3, entries 1–3). To eliminate cryogenic conditions, an alternative oxidation with TFAA (supplanting oxalyl chloride) was pursued.^32^ The reaction of **14** with TFAA/DMSO in the presence of TEA at −20 °C yielded **8** in 67 A % yield (GCMS-TIC) (Table 3, entry 4). Unfortunately, optimization of this oxidation by varied conditions (increase of 1 equiv of TFAA, reaction time, temperature) resulted in no improvement but rather yielded low purity profiles of **8**. Oxidation of **14** under the Dess–Martin conditions^33^ effectively produced **8** in 87 A % (GCMS-TIC) (Table 3, entry 5). Ley–Griffith oxidation [morpholine N-oxide (NMO)/tetrapropylammonium perruthenate (TPAP)] was also effective in the oxidation of **14** to **8** (Table 3, entry 6).^34^ However, neither Dess–Martin oxidation nor Ley–Griffith oxidation is process-friendly due to safety concerns and the high cost issues of the reagent and catalyst. Finally, Albright–Goldman oxidation was identified to be a possible scalable condition to yield **8** (Table 3, entries 7–13).^35^ Under a standard Albright–Goldman oxidation, the reaction of **14** with DMSO (7 equiv) and Ac_2_O (11 equiv) at 50 °C afforded **8** in 79 A % (GCMS-TIC) (Table 3, entry 7). Excess of Ac_2_O and DMSO were required to achieve a >80 A % (GCMS-TIC) of **8**. Attempts to decrease the amount of Ac_2_O and DMSO with or without solvents resulted in low conversion (30–60 A %, GCMS-TIC) (Table 3, entries 8–10). It is worth mentioning that the formation of the Pummerer rearrangement byproduct (**BP**) was inevitable under these Albright–Goldman conditions, and the amount of the Pummerer rearrangement byproduct was dependent on the reaction time. As shown in Entry 7 (Table 3), a high level of the Pummerer rearrangement byproduct (**8/BP** = 1/6) was observed after 14h. An increase in the equivalents of Ac_2_O and DMSO dramatically shortened the reaction time, resulting in a low level of the byproduct.^36^ For example, a reaction of **14** with DMSO (11 equiv) and Ac_2_O (14 equiv) at 50 °C was complete within 7 h, delivering **8** in 86 A % (GCMS-TIC), and the ratio of **8**/**BP** was 1/2 (Table 3, entry 11). A mixture of DMSO (14 equiv)/Ac_2_O (14 equiv) allowed the completion of the reaction within 4 h, and the ratio of **8**/**BP** was 1/1 (Table 3, entry 12). The ratio of **8**/**BP** was increased to 2/1 when performing the oxidation with DMSO (19 equiv)/Ac_2_O (14 equiv), in which the reaction was complete within 2 h (Table 3, entry 13). After workup (basification with saturated NaHCO_3_ and extraction with ethyl acetate), the resulting organic mixture was distilled to afford pure **8** in an ∼70% isolated yield. Ultimately, this optimal Albright–Goldman oxidation (DMSO (19 equiv)/Ac_2_O (14 equiv)) was used for the scale-up of **8** (vide infra).

**Table 3:**
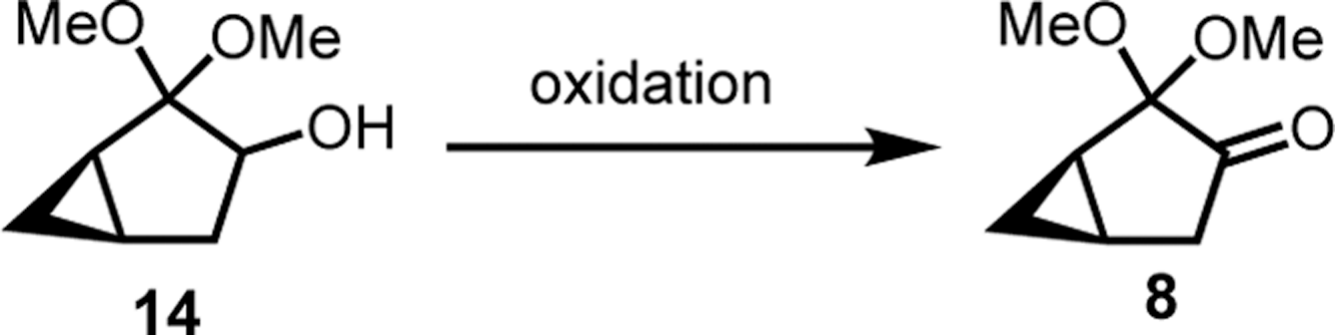
Oxidation Trials and Optimization to Convert Alcohol **14** into Ketone **8**

entry^a^	variations of the baseline condition	8 (A %, GCMS-TIC)^b^
1	baseline condition: (COCl)_2_ (1.2 equiv), TEA (5 equiv), DMSO (2.4 equiv), DCM (15 V), –60 °C, 1 h	92^c^
2	(COCl)_2_ (1.2 equiv), TEA (5 equiv), DMSO (2.4 equiv), DCM (15 V), –20 °C, 1 h	complex
3	(COCl)_2_ (1.2 equiv), TEA (5 equiv), DMSO (2.4 equiv), DCM (15 V), 0 °C, 1 h	complex
4	TFAA (1.2 equiv), DMSO (1.1 equiv), TEA (3.3 equiv), DCM (10 V), –20–15 °C, 1 h	67^d^
5	DMP (1.25 equiv), 25 °C, DCM (5 V), 1 h	87
6	NMO (2.7 equiv), TPAP (0.09 equiv), 25 °C, 24 h	61^e^
7	Ac_2_O (11 equiv), DMSO (7 equiv), 50 °C, 14 h	79 (**8/BP** = 1/6)^f^
8	Ac_2_O (2 equiv), DMSO (7 equiv), 80 °C, 2 h	59^d^
9	Ac_2_O (2 equiv), DMSO (2 equiv), 50 °C, 18 h, toluene	30^d^
10	Ac_2_O (2 equiv), DMSO (2 equiv), 80 °C, 16 h, toluene	50^d^
11	Ac_2_O (11 equiv), DMSO (14 equiv), 50 °C, 7 h	86 (**8/BP** = 1/2)^f^
12	Ac_2_O (14 equiv), DMSO (14 equiv), 50 °C, 4 h	89 (**8/BP** = 1/1)^f^
13	**Ac**_**2**_**O(14 equiv),DMSO(11 equiv),50 °C,1.5–2 h**	89 (**8/BP** = 2/1)^f^

a Typical procedure: **14** (purified, 0.5 g), oxidant, conditions as shown in the table.

b A % of IPC was measured by GCMS-TIC.

c Isolated yield: 72%, after column purification.

d Major starting material **14** remained.

e Isolated yield: 52%, after column purification.

f **BP**: (methylthio)methyl acetate generated from the Pummerer rearrangement, the ratio was obtained by crude ^1^HNMR.

Notably, a basification process is used to terminate the undesired Pummerer rearrangement. This treatment requires at least 50 V of saturated NaHCO_3_ solution to neutralize the reaction mixture. As a result, up to a 70 V maximum operating volume was needed, which becomes problematic on scale. To accomplish a more practical workup process for the Albright–Goldman oxidation, stronger and more concentrated bases were examined. NaOH was first examined to replace the NaHCO_3_ for basification. The basification proceeded smoothly with 5 M NaOH, and the total quenching volume was decreased to 14 V. It was critical to control the temperature <−10 °C to avoid unknown impurities being generated during the quenching process. However, NaOH-based quenching process is strongly exothermic and it will be challenging to control the low temperature at scale. Lowering the concentration of NaOH to 1 or 2 M facilitated the low temperature control and mitigated the side reactions but the total operating volumes increased to 65–130 V. Switching to a 28 wt % NH_4_OH solution, 10 V of the basic solution allowed for the basification; unfortunately, imine impurities were observed by ^1^HNMR. Advantageously, cold water was found to be a good choice to quench the reaction mixture. The reaction mixture was treated with ice water (10 V), followed by extraction with ethyl acetate, thereby allowing the partitioning of **8** and Ac_2_O into the organic phase. This process terminated the Pummerer rearrangement. There are two major advantages to using water compared to basic quench: 1) the water quench mitigates unexpected side reactions during the workup; 2) the water-miscible DMSO allows separation from Ac_2_O after ethyl acetate extraction and eliminates the Pummerer rearrangement. The identification of cold water for the workup dramatically reduced the operating volume and the subsequent purification process became straightforward. For example, after quenching with ice water (10 V), the reaction mixture was extracted with ethyl acetate (10 V × 3), and **8**, **BP,** Ac_2_O, and a small amount of DMSO and AcOH were separated in the organic layer. Further washing the organic layer with NaHCO_3_ (9 V × 3) removed DMSO and AcOH and the resulting organic layer was subjected to distillation to afford **8** in a good yield and excellent purity.

With the optimized condition for synthesis of **8** in hand, scale-up of the process to a hundred-gram scale was demonstrated. As summarized in Table 4 (batch 1–3), this 2-step protocol delivered compound **8** in an overall 45–56% isolated yield with 95–97 A % purity (**BP** < 1 A % and **14a** < 4 A %) (GCMS-TIC). The purity of **8** was further improved up to >99 A % (GCMS-TIC) by precipitation of the compound **8** from hexanes at −40 °C.

**Table 4:**
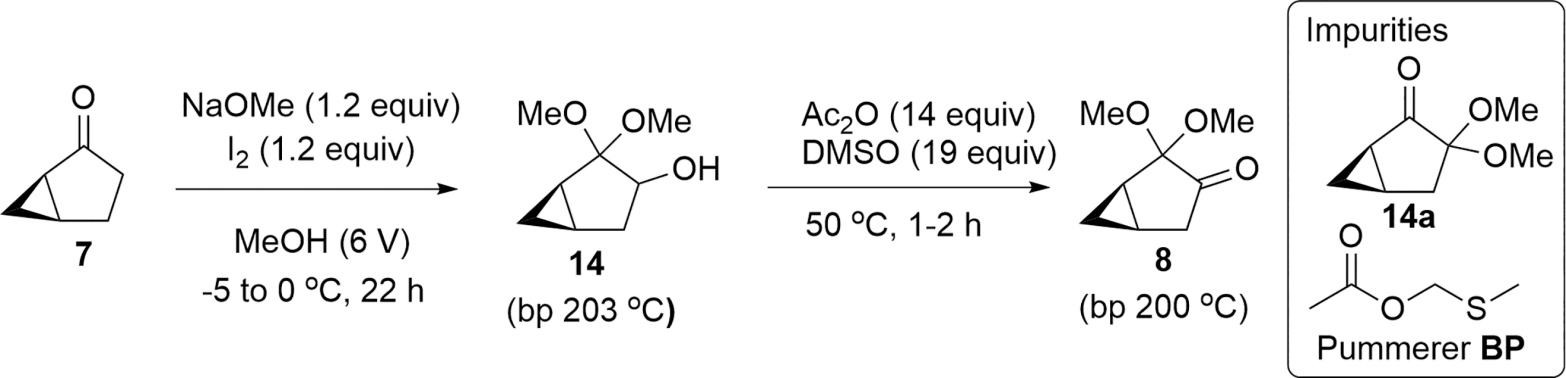
Scale-Up Summary of Two-Step Synthesis of **8** from Bicyclic Ketone **7**

		A % (GCMS-TIC)^a^						8 (after distillation)^c^			
		hydroxylation			A–G oxidation^b^						
batch	**7** (g)	**14**	**14a**	**7**	**8**	**14a**	**BP**	amount (yield)^d^	purity (wt %)^e^	purity (A %)^f^	ee (%)^g^
1	106	91	6	<1	80	5	15	77 g (45%)^h^	94	95	100
2	100	94	6		68	4	28	91 g (56%)	97	95	100
3	90	92	5	1	75	4	21	77 g (53%)	98	97	100

a TIC A % was measured by GCMS; all data was obtained from crude reaction mixture of each step after aqueous workup.

b A–G oxidation: Albright–Goldman oxidation.

c Distilled at 50 °C under 7 Torr to remove Ac_2_O and at 60 °C under 1–2 Torr to afford **8**.

d Corrected two-step isolated yield.

e Wt % was obtained by qNMR with mesitylene as an internal standard.

f A % was obtained by GCMS-TIC; A % of Pummerer **BP** < 1 A % and possible isomer **14a** ∼3–4 A %; A % of **8** was improved up to >99 A % (GCMS-TIC) after precipitation from hexanes at −40 °C.

g Enantiomeric excess (ee) was measured by chiral GC.

h The product loss during distillation resulted in a low isolated yield on this batch.

## Conclusions

In conclusion, an asymmetric synthesis of (1*R*,5*R*)-2,2-dimethoxybicyclo[3.1.0]hexan-3-one has been disclosed for the first time. This approach employed an inexpensive commodity ((*R*)-epichlorohydrin) as the starting material, featured a 4-step telescoped bicyclic ketone synthesis, an I_2_-promoted hydroxylation, and an Albright–Goldman oxidation, offering (1*R*,5*R*)-2,2-dimethoxybicyclo[3.1.0]hexan-3-one in an overall 22–31% isolated yield with >95 A % (GCMS-TIC) chemical purity and excellent enantiomeric purity (ee >99%). The process allows for practical isolation steps throughout and has been successfully demonstrated on a hundred-gram scale input of **7**. It is hoped that this scalable approach to (1*R*,5*R*)-2,2-dimethoxybicyclo[3.1.0]hexan-3-one will inspire further inquiry and find its application in further efforts toward cost-effective synthesis of lenacapavir.

## Experimental Section

### General Information

Reagents and solvents were obtained from commercial suppliers and used as received unless otherwise indicated. (*R*)-Epichlorohydrin (99%) was purchased from Oakwood Chemical and allylmagnesium chloride solution (2.0 M in THF) was purchased from Sigma-Aldrich. Reactions were conducted in oven-dried (120 °C) glassware, which was assembled while hot and cooled to ambient temperature under an inert atmosphere. All reactions were conducted under an inert atmosphere (N_2_) unless otherwise noted. Reactions were monitored by TLC (precoated silica gel 60 F254 plates, EMD Chemicals), GCMS or chiral-column GC using various methods. TLC was visualized with UV light or by treatment with phosphomolybdic acid (PMA), ninhydrin, and/or KMnO_4_. Flash chromatography was performed on a Teledyne ISCO Combi-Flash NEXTGEN 300+ and/or a Biotage Isolera using solvents as indicated. HRMS was recorded using PerkinElmer Axion 2 ToF MS, ionization mode: positive with scan range: 100–1000 *m*/*z*, flight tube voltage: 8 kV, spray voltage: 3.5 kV, solvent: methanol. ^1^HNMR and ^13^CNMR spectra were routinely recorded on a Bruker Avance III HD Ascend 600 MHz spectrometer. The NMR solvents used were CDCl_3_ or CD_3_CN as indicated. Tetramethylsilane (TMS) was used as an internal standard. Coupling constants J are reported in hertz (Hz). The following abbreviations were used to designate signal multiplicity: s, singlet; d, doublet; t, triplet; q, quartet, p, pentet; dd, doublet of doublets; ddd, doublet of doublet of doublets; dt, double of triplets; ddt, doublet of doublet of triplets; m, multiplet; br, broad. 1,3,5-trimethoxybenzene and triphenylmethane were used as internal standards for quantitative ^1^H NMR.

### Telescoped Synthesis of 7 (Based on the Entry 2 in Table 1)

#### Synthesis of (*R*)-1-Chlorohex-5-en-2-ol (**11**)

(*Caution: Both compounds***10***and***11***are classified as hazardous substances. All operations involving these compounds must be conducted in a fume hood and appropriate personal protective equipment must be worn at all times to ensure safety and minimize exposure risks.)* THF (150 mL, 1 V) and (*R*)-epichlorohydrin **10** (148 g, 1.6 mol, 1 equiv) were charged to a 5L ChemRxnHub reactor under a nitrogen atmosphere. This mixture was cooled at −25 °C (internal temperature was −14.5 °C) using a Huber circulating chiller. When the internal temperature achieved −14.5 °C, allylmagnesium chloride (800 mL, 1.6 mol, 1 equiv, 2 M in THF) was added using a peristaltic pump at a flow rate of 5 mL/min, maintaining the internal temperature below −5.0 °C. After addition, this mixture was stirred at the same temperature for an additional 1 h. After completion of the reaction (monitored by GC–MS), methanol (162 mL, 4.0 mol, 2.5 equiv) was added dropwise, keeping the internal temperature below 0 °C, followed by addition of HCl (1.6 L, 2 M, 2.0 equiv) at 0 °C. The circulating cooling system was turned off and MTBE (740 mL) was added. The organic layer was collected and washed with HCl (300 mL, 2 M) and water (300 mL), respectively. This resulting organic layer (1360 mL corresponding to 1150 g) gave an in-solution yield of 99% (corrected weight 215.3 g) **11** assayed by GC–MS (19 wt %), with 95 A % GCMS-TIC (excluding solvent peaks etc.), ee: 99.9% and containing 0.7 A % of dichlorohydrin and 1.8 A % of epoxide **12** by GCMS-TIC. The crude compound **11** was used in the next step without further purification. A small amount of a concentrated solution was used for ^1^H NMR analysis.

^**1**^**H NMR** (600 MHz, CDCl_3_): δ 5.78 (ddt, *J* = 17.0, 10.2, 6.7 Hz, 1H), 5.01 (ddd, *J* = 17.1, 3.4, 1.7 Hz, 1H), 4.94 (ddd, *J* = 10.2, 3.0, 1.3 Hz, 1H), 3.55 (dd, *J* = 11.1, 3.9 Hz, 1H), 3.44 (dd, *J* = 11.1, 6.7 Hz, 1H), 3.05 (d, *J* = 5.7 Hz, 1H), 2.31 (s, 2H), 2.25–2.08 (m, 2H), 1.63–1.54 (m, 2H). **MS-EI** (*m*/*z*) (M^•+^): 134.

#### Synthesis of *R*-(+)-1,2-Epoxy-5-hexene (**12**)

A solution of chlorohydrin **11** in MTBE (1150.47 g, 19 wt %, ∼ 1.4 L) was charged in a 5 L ChemRxnHub reactor, and then an aqueous solution of NaOH (960 mL, 1.2 equiv, 2 N) was added. The mixture was heated to 50 °C and stirred for 2 h. After completion of the reaction, the organic layer was collected and washed with water (250 mL × 4) until the aqueous layer had pH = 7. The organic layer (∼1.0 L, 807 g, 19 wt % by qNMR, KF = 1.4%, 98.9 A % by GCMS) was then used for drying. The above MTBE solution (∼1.0 L, 807 g, KF: 1.4%) was charged to a 2 L RBF with a Dean–Stark trap. The solution was refluxed at 70 °C for 16 h and about 10 g of water was removed. The KF of the resulting MTBE solution was measured to be 0.19%. This resulting organic layer (776 g) gave an in-solution yield of 87% (corrected weight 136 g, over two steps from **10**) epoxide **12** assayed by GC–MS (17.6 wt % purity by qNMR), with 98.8 A % GCMS-TIC (excluding solvent peaks etc.) and contained 1.2 A % (GCMS-TIC) of epichlorohydrin . The crude compound **12** was used for the next step without further purification. A small amount of concentrated solution was used for ^1^H NMR analysis.

^**1**^**H NMR** (600 MHz, CDCl_3_): δ 5.81–5.72 (m, 1H), 5.02–4.87 (m, 2H), 2.88–2.81 (m, 1H), 2.67 (t, *J* = 4.5 Hz, 1H), 2.40 (dd, *J* = 5.0, 2.7 Hz, 1H), 2.20–2.07 (m, 2H), 1.61–1.48 (m, 2H). **MS-EI** (*m*/*z*) (M^•+^): 98.

#### Synthesis of (1*S*,2*R*,5*R*)-Bicyclo[3.1.0]hexan-2-ol (**13**)

A 5 L ChemRxnHub reactor was charged with a dried solution of epoxide **12** in MTBE (776 g, 17.6 wt %). TMP (118 mL, 0.5 equiv) was added to the reactor. This mixture was cooled at −10 °C (internal temperature was −9.9 °C) using a Huber circulating chiller. When the internal temperature achieved −9.9 °C, *n*-butyllithium (612 mL, 1.5 mol, 1.1 equiv, 2.5 M in hexane) was added using a peristaltic pump with a flow rate of 4.0 mL/min, maintaining the internal temperature below −5.0 °C. After the addition, this mixture was stirred at the same temperature for an additional 1 h. After completion of the reaction, HCl (745 mL, 3 M, 1.6 equiv) was added dropwise, keeping the internal temperature below 25 °C. The circulating cooling system was turned off, and the aqueous layer was drained (pH = 10). Then, more HCl (235 mL, 3 M, 0.5 equiv) was added to wash the organic layer (pH = 1). The combined aqueous layer was charged back to the reactor and extracted twice with 5 and 2.5 V of MTBE (685 and 340 mL). This combined organic layer (2640 mL) was concentrated at 40 °C under vacuum (200–340 mbar) to about 5 V. This resulting organic layer (699.09 g, 900 mL) afforded bicycloalcohol **13** with an in-solution yield of 72% (corrected weight 98.08 g) by qNMR (14.03 wt %, 89.2 A % (excluding solvent peaks etc.) or 11.6% purity by GC–MS). The crude compound **13** was used in the next step without further purification. A small amount of compound **13** was purified by distillation for analytical purposes (under 455 to 310 mbar vacuum at 70 °C to remove MTBE and further distillation at 45 °C and 1–6 mbar to afford the desired compound **13** as an oil).

^**1**^**H NMR** (600 MHz, CDCl_3_): δ 4.21 (d, *J* = 4.7 Hz, 1H), 1.97–1.72 (m, 2H), 1.65 (dd, *J* = 12.3, 8.3 Hz, 1H), 1.53 (dd, *J* = 14.5, 8.3 Hz, 1H), 1.44–1.37 (m, 1H), 1.31 (ddd, *J* = 12.4, 8.2, 4.8 Hz, 2H), 0.41 (dd, *J* = 7.7, 5.7 Hz, 1H), 0.00 (dd, *J* = 7.9, 4.2 Hz, 1H). ^**13**^**C{**^**1**^**H} NMR** (151 MHz, CDCl_3_): δ 74.5, 30.4, 26.9, 24.5, 16.1, 6.9. **IR (ATR)** ν_**max**_ = 3328.5, 2935.3, 2870.1, 1328.8, 1177.8, 1101.4, 1043.7, 984.0, 870.3, 808.8, and 717.5 cm^–1^. **MS-EI** (*m*/*z*) (M^•+^): 98. [**α**]_**D**_^20^ (deg·mL·g^–1^·dm^–1^) **(**MeOH (10 mg/mL) at 20 °C under 589 nm): +23.96.

#### Synthesis of (1*S*,5*R*)-Bicyclo[3.1.0]hexan-2-one (**7**)

A 5L ChemRxnHub reactor was charged with a solution of bicycloalcohol **13** in MTBE (699.09 g, 14.03 wt %). To the reactor was added a solution of K_2_HPO_4_ (366.3 g, 2.1 mol, 1.5 equiv) in water (555 mL, 22 equiv) followed by TEMPO (5.44 g, 34.6 mmol, 0.025 equiv). This mixture was cooled at −5 °C (internal temperature was −3.8 °C) using a Huber circulating chiller. When the internal temperature achieved −3.8 °C, 10% sodium hypochlorite solution (1,3 L, 2.1 mol, 1.5 equiv) was added using a peristaltic pump with a flow rate of 12 mL/min, maintaining the internal temperature below 5.0 °C. After the addition, the circulating cooling system was turned off, and when the internal temperature achieved 20 °C, the reaction was stirred for an additional 3 h. After completion of the reaction, sodium sulfite solution (61.1 g, 0.35 equiv in 280 mL of water) was added, keeping the internal temperature below 30 °C. The organic layer was separated and the aqueous layer was extracted twice with 5 and 2.5 V MTBE (685 and 340 mL). This combined organic layer (1840 mL, 87 A %) was concentrated at 40 °C under vacuum (200–340 mbar) to remove MTBE. This resulting residue 196.33 g (210 mL) gave an in-solution yield of 94.1% (90.4 g) bicyclo ketone **7** assayed by qNMR (46.06 wt %).

#### Purification

The resulting crude material was distilled at 25–40 °C under vacuum (4–1.8 Torr) with a vapor temperature between 30 and 40 °C to afford the desired bicyclo ketone **7** (91.55 g, 4-step overall yield: 55% based on the purity, purity: 92 A % by GCMS-TIC and 94 wt % by qNMR, *ee*: 100%).

^**1**^**H NMR** (600 MHz, CDCl_3_): δ 2.19–1.94 (m, 5H), 1.78–1.70 (m, 1H), 1.22–1.14 (m, 1H), 0.96–0.88 (m, 1H). ^**13**^**C{**^**1**^**H} NMR** (151 MHz, CDCl_3_): δ 215.2, 31.4, 27.4, 22.6, 21.6, 13.5. **MS-DART**(*m*/*z*) (MH^+^): 97.1. **IR (ATR)** ν_**max**_ = 3328.5, 2935.3, 2870.1, 1328.8, 1177.8, 1101.4, 1043.7, 984.0, 870.3, 808.8, 717.5 cm^–1^. **GC** (RT-GammaDEXsa (30 m × 0.25 mm × 0.25 μm), column flow = 0.56 mL/min, H_2_ flow = 35.0 mL/min, air flow = 450 mL/min) *t*_R_ = 19.1 min (100%), 21.0 min (0%). **Specific rotation**: [α]_D_^20^ = +20.79 (deg·mL·g^–1^·dm^–1^) (measured in MeOH (10 mg/mL) at 20 °C under 589 nm).

#### Typical Procedure of Synthesis of (1*R*,5*R*)-2,2-Dimethoxybicyclo[3.1.0]hexan-3-ol (**14**)

A 2 L ChemRxnHub reactor was charged with MeONa/MeOH (563.3 g, 596 mL, 25 wt %, 2.52 equiv, 2.607 mol) and cooled to −10 °C under N_2_. Iodine (315.0 g, 1.2 equiv, 1.241 mol) was added to the reactor once the internal temperature reached −5 °C. A slight exotherm was noticed (2–10 °C) during this operation. The resulting solution was allowed to stir for 15 to 20 min at −5 °C to −10 °C. Then, (1*R*,5*S*)-bicyclo[3.1.0]hexan-2-one **7** (105.80 g, 94 wt %, 1 equiv, 978 mmol) was added dropwise using a peristaltic pump (2 mL/min) maintaining NMT 0 °C internal temperature. The reaction mixture was stirred at 0 °C overnight. After completion of the reaction (monitored by GCMS and ^1^H NMR), the reaction mixture was concentrated under a vacuum (45 °C) to remove the MeOH. The resulting residue was dissolved in DCM (1000 mL, 10 V) and stirred for 10 min. Water (200 mL, 2 V) was added, and the mixture was stirred for another 10 min. The DCM layer was separated, and the aqueous layer was extracted with DCM (300 mL × 1, 3 V). The combined DCM solution was charged to the reactor and washed with saturated Na_2_SO_3_ solution (200 mL × 2 V) and brine (200 mL, 2 V). The DCM layer was collected (**14**: 91 A %; **14a**: 6 A %; **7**: <1 A % by GCMS-TIC) and concentrated *in vacuo* to afford the crude product **14** (160.35 g, 82% qNMR purity, 80% yield; 2 A % byproduct **14a**) as a pale-yellow oil. The crude product was used in the next step, with further purification. A small amount of compound **14** was purified by distillation for analytical purpose (the crude of **14** was distilled at 40 °C under 1 mbar to afford the desired compound **14** as an oil).

^**1**^**H NMR** (600 MHz, CDCl_3_): δ 3.97 (d, *J* = 7.1 Hz, 1H), 3.44 (s, 3H), 3.25 (s, 3H), 2.37 (s, 1H), 2.24–2.15 (m, 1H), 1.78 (dd, *J* = 14.1, 0.8 Hz, 1H), 1.53–1.44 (m, 1H), 1.38 (dd, *J* = 8.8, 5.0 Hz, 1H), 0.82 (dd, *J* = 9.0, 4.2 Hz, 1H), 0.58 (ddt, *J* = 8.5, 5.0, 1.2 Hz, 1H). ^**13**^**C{**^**1**^**H} NMR** (151 MHz, CDCl_3_) δ: 110.3, 72.3, 51.3, 49.2, 34.1, 21.8, 14.9, 8.5. **IR (ATR)** ν_**max**_ = 3475.7, 2942.7, 2834.6, 1448.1, 1366.1, 1341.8, 1142.4, 1049.2, 1030.6, 978.4, 911.3, 814.4 cm^–1^. **HRMS (ESI)***m***/***z***:** calcd for C_8_H_14_ O_3_·Na^+^ = [M + Na]^+^ 181.0841; found, 181.0844.

#### Typical Procedure of Synthesis of (1*R*,5*R*)-2,2-Dimethoxybicyclo[3.1.0]hexan-3-one (**8**)

A 5 L ChemRxnHub reactor was equipped with a thermocouple, baffle, N_2_ flow, and a bleach trap. DMSO (1.2 kg, 1.1 L, 19 equiv, 15.8 mol) and (1*R,*5*R*)-2,2-dimethoxybicyclo[3.1.0]hexan-3-ol (1*R,*5*R*)-2,2-dimethoxybicyclo[3.1.0]hexan-3-ol **14** (160.3 g, 832 mmol, 82% purity) were charged to the reactor at 25 °C. Acetic anhydride (1.2 kg, 1.1 L, 14 equiv, 11.6 mol) was added at the same temperature. The reaction mixture was heated to 50 °C and stirred for 1.5 h under N_2_. After completion of the reaction (once **14** was <5 A % by GC–MS and ^1^H NMR), the reaction mixture was cooled to 0 °C and diluted with water (1.6 L, 10 V) and EtOAc (1.6 L, 10 V). The mixture was stirred at 0 °C for 30 min. The water layer was separated and extracted with EtOAc (800 mL × 2). The combined EtOAc layer was washed with sat. NaHCO_3_ (1000 mL × 3) to pH = 6–7 and then brine (1 L). The resulting EtOAc layer (**8**: 80 A %; **14a**: 5 A %; **BP**: 15 A % by GCMS-TIC) was concentrated in the reactor under 300–50 Torr at 50 °C to afford the crude **8** as a pale-yellow liquid (189 g, 40 wt % by GC).

The crude product was further purified by vacuum distillation using a 12 in. Vigreaux column and a JKEM vacuum controller. The first fraction was collected at 50 °C under 35–7 Torr, to purge a mixture of EtOAc, acetic anhydride, and Pummerer BP as the first fraction (69 g). The resulting residue was distilled at 60 °C under 3 Torr to afford **BP** as the major fraction (5.2 g). The resulting residue was distilled at 60 °C under 1–2 Torr to afford **8** (77 g, two-step overall yield: 45%, 94 wt % by qNMR, 95 A % by GCMS-TIC, containing <1 A % the Pummerer **BP** and 3–4A % **14a** by GCMS-TIC). The obtained **8** was dissolved in hexanes (390 mL, 5 V) and cooled to −40 to −45 °C with overhead stirring and **8** was formed as an amorphous solid. Once precipitation of **8** was complete, the supernatant was suctioned through a 20 μm glass inlet filter via a peristaltic pump at 20 mL/min until dry. The residue was quantitatively collected with MTBE and concentrated *in vacuo* to yield **8** as a pale-yellow oil (68 g, 98 wt % by qNMR, 97 wt % by GC, 100 A % by GCMS-TIC, two-step overall yield after precipitation: 41%).

^**1**^**H NMR** (600 MHz, CDCl_3_): δ 3.19 (s, 3H), 3.01 (s, 3H), 2.54 (ddd, *J* = 18.8, 5.5, 2.2 Hz, 1H), 2.04 (d, *J* = 18.7 Hz, 1H), 1.49 (td, *J* = 7.9, 4.2 Hz, 1H), 1.42–1.34 (m, 1H), 0.71 (dq, *J* = 8.2, 2.1 Hz, 1H), −0.01 (dt, *J* = 6.3, 4.3 Hz, 1H). ^**13**^**C{**^**1**^**H} NMR** (151 MHz, CDCl_3_): δ 207.7, 102.9, 50.9, 50.5, 38.9, 19.2, 9.3, 8.9. **IR (ATR)** ν_**max**_ = 2994.9, 2946.5, 2909.2, 1755.6, 1092.1, 1067.9, 1043.7, 1017.6, 997.1, 915.1, 810.7 cm^–1^. **HPLC** (CHIRALPAK IG-3 SFC, CO_2_/MeOH = 95/5, flow rate = 2.0 mL/min, detector wavelength = 210 nm) *t*_R_ = 2.7 min (100%), 3.0 min (0%). **Specific rotation**: [α]_D_^20^ = +57.13 (deg·mL·g^–1^·dm^–1^) (measured in MeOH (10 mg/mL) at 20 °C under 589 nm). **HRMS (ESI)***m***/***z***:** calcd for C_8_H_12_O_3_·Na^+^ = [M + Na]^+^ 179.0684; found, 179.0680.

## Supporting Information Available

Additional experimental details and analytical methods (PDF)
