# Supplementary material for: Development of a Scalable Synthetic Route to (1R,5R)-2,2-Dimethoxybicyclo[3.1.0]hexan-3-one: An Important Intermediate in the Synthesis of Lenacapavir
Source: Org Process Res Dev. 2025 Feb 26;29(3):846–55. doi: 10.1021/acs.oprd.4c00527 (PMC11934127; doi:10.1021/acs.oprd.4c00527)
Supplement: Supplementary file 1 — op4c00527_si_001.pdf [file op4c00527_si_001.pdf]

Development of a scalable synthetic route to (*1R,5R*)-2,2-dimethoxybicyclo[3.1.0]hexan-3-one: an important intermediate in the synthesis of lenacapavir

*Aline Nunes De Souza,[‡] Nagaraju Sakkani,[‡] Daryl Guthrie,[‡] Rajkumar Lalji Sahani,[‡] John M. Saathoff,[‡] Samuel R. Hochstetler, Justina M. Burns, Saeed Ahmad, G. Michael Laidlaw, B. Frank Gupton, Douglas A. Klumpp, Limei Jin**

Medicines for All Institute, Virginia Commonwealth University, Richmond, VA, 23284-3068.

[‡]These authors contributed equally

*Email: jinl3@vcu.edu

Medicines for All Institute, Virginia Commonwealth University, Richmond, VA, 23284-3068.

GENERAL METHODS

Reagents and solvents were obtained from commercial suppliers and used as received unless otherwise indicated. Where applicable, reactions were conducted in oven-dried (120°C) glassware, which was assembled while hot, and cooled to ambient temperature under an inert atmosphere. Reactors were pre-rinsed with reaction solvent and subjected to evacuation/back-fill cycles (3x) as necessary. All reactions were conducted under an inert atmosphere (N_2) unless otherwise noted. Reactions were monitored by TLC (precoated silica gel 60 F254 plates, EMD Chemicals), Agilent GC-MS, chiral Agilent GC-FID or chiral SFC using various methods. TLC was visualized with UV light or by treatment with Phosphomolybdic acid (PMA), ninhydrin, and/or $KMnO_4$. ^{1}H NMR and ^{13}C NMR spectra were routinely recorded on Bruker Avance III HD Ascend 600 MHz spectrometer. All chemical shifts are reported in parts per million (ppm) relative to residual $CHCl_3$

(7.26 ppm for ^{1}H, 77.16 ppm for ^{13}C) or tetramethylsilane (0.0 ppm for ^{1}H and ^{13}C). Coupling constants J are reported in hertz (Hz). The following abbreviations were used to designate signal multiplicity: s, singlet; d, doublet; t, triplet; q, quartet, p, pentet; dd, doublet of doublets; ddd, doublet of doublet of doublets; dt, double of triplets; ddt, doublet of doublet of triplets; m, multiplet; br, broad.

GC-MS Methods. Formation of **8** through **10** was monitored via GC-MS (Agilent 6890 GC-5977 MSD). This method was used for monitoring and quantitation for all molecules in the route with the exception of **12** which was quantitated using the method noted below. The column used was an Agilent J&W HP-1 GC Column, 30 m, 0.32 mm, 5.00 µm. The inlet was set to 250 °C. A split ratio of 50:1 was used with an injection volume of 1.0 µL. The column flow rate was 1.4 mL/min with helium as the carrier gas and an inlet pressure of 7.87 psi. The oven was initially set to 50 °C for 3 minutes, linearly ramped to 225 °C at 25 °C/min and held for an additional 5 minutes.

Structures & IDs:

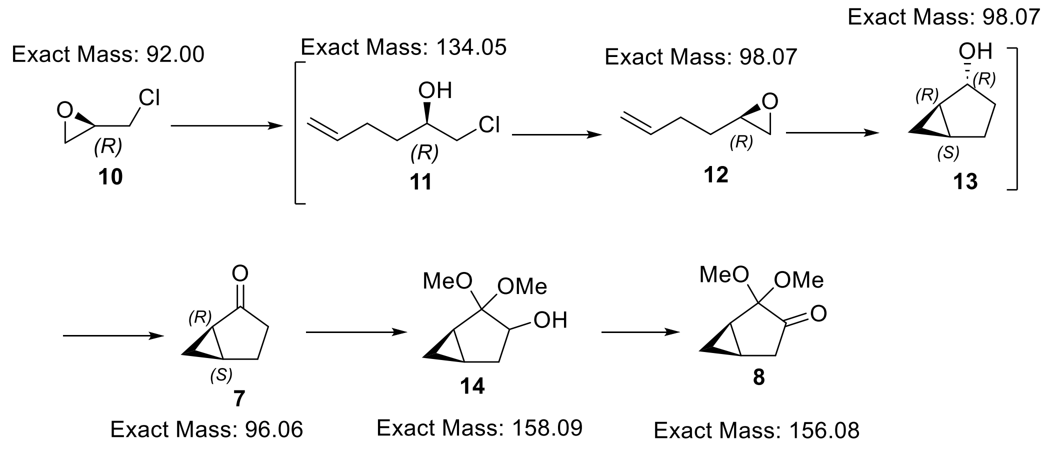

<u>Instrument Type:</u> Agilent 6890 gas chromatograph (GC) with a 5977 mass spectrometer detector (MSD)

<u>Conditions:</u>

<u>Column:</u> J&W HP-1 GC Column, 30 m, 0.32 mm, 5.00 µm

<u>Inlet Pressure:</u> 7.87 psi	<u>Split Ratio:</u> 50 : 1	<u>Split Flow:</u> 70 mL/min
<u>Column flow:</u> 1.4 mL/min	<u>Injection Temp:</u> 250 °C	<u>Injection volume:</u> 1 µL

Total Flow: 74.11 mL/min   Solvent Delay: 3.5 min   Runtime: 15 min

Temperature Program:

Time (min)	Ramp (°C/min)	Temp (°C)	Hold (min)
Initial	-	50	3
-	25	225	5

MS Parameters:

Transfer Line Temp (°C)	250
Source Temp (°C)	230
Quad Temp (°C)	150
Electron Energy (eV)	70
Mass Range	30-1000

Sample preparation:

Prepare solutions at approximately 0.5 - 1 mg/mL in acetonitrile or methanol for qualitative analysis.

**Retention Times**		
**Compound**	***m/z***	**Time (min)**
**MTBE**	73	4.3
**Hexa-1,5-diene**	81, 67	4.6
**THF**	72	5.2
**10**	62, 57	6.3
**12**	97, 67	7.3
**Dichlorohydrin**	79	8.3
**13**	97	8.4
**7**	96	8.9
**11**	116, 81	9.2
**8**	128, 125	10.6
**14**	141, 117, 88	10.7

Representative Chromatogram

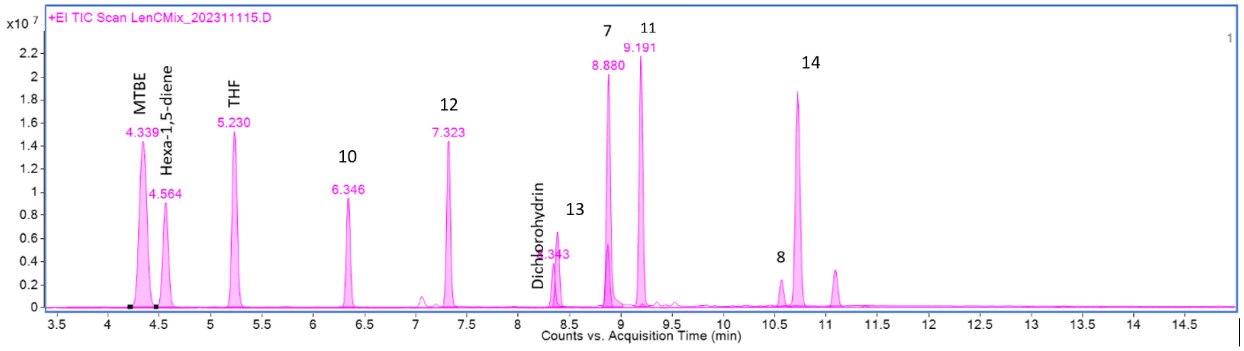

Mass spectra:

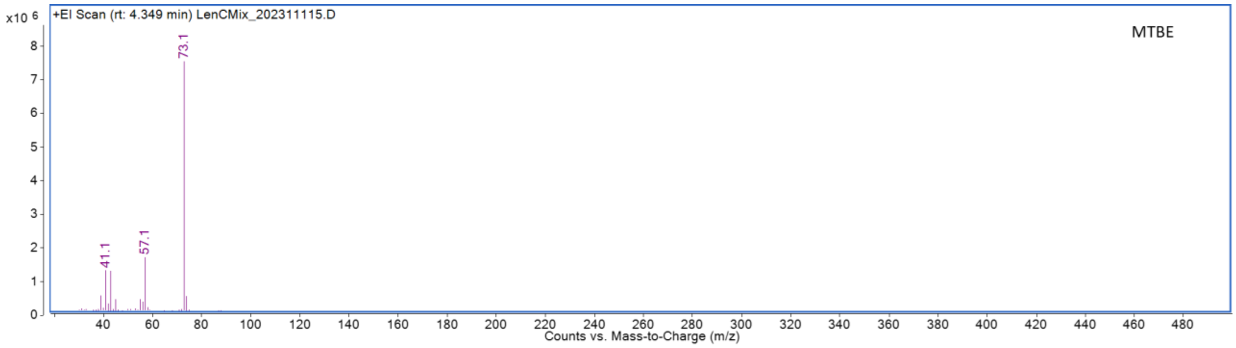

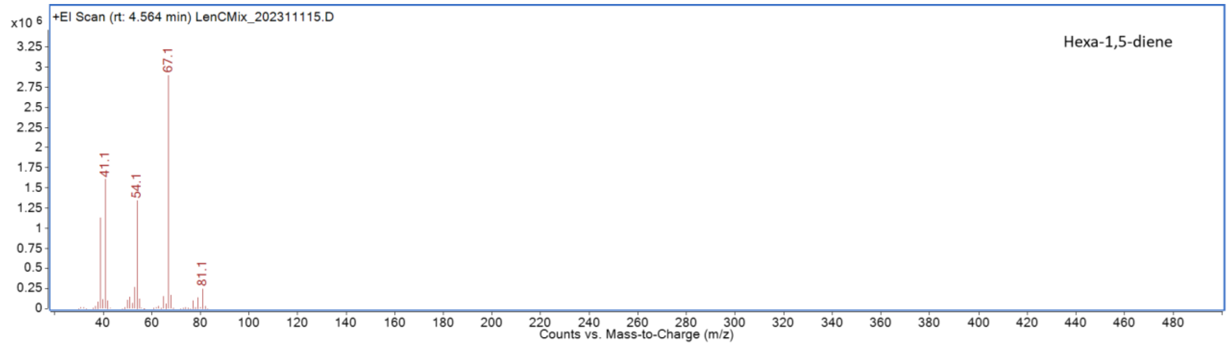

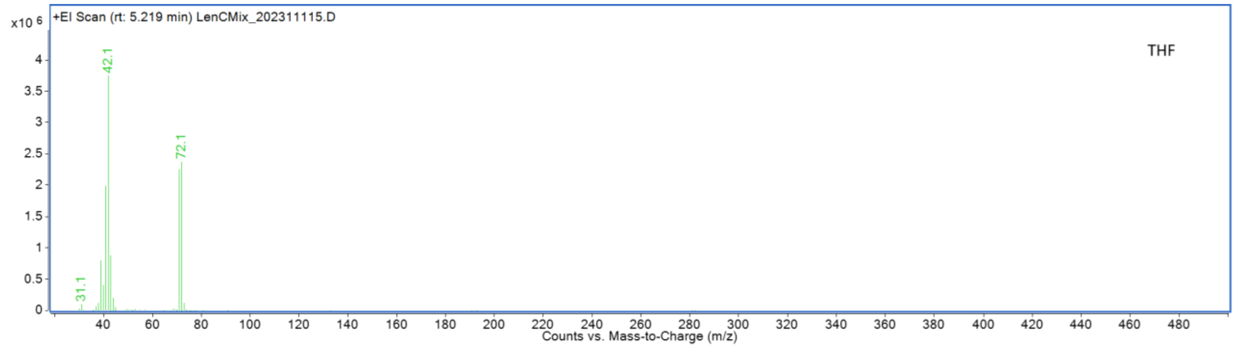

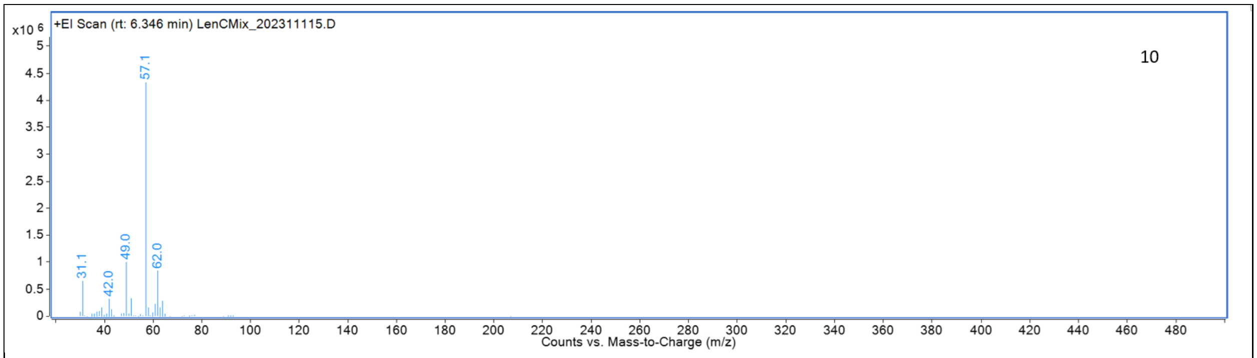
+EI Scan (rt: 6.346 min) LenCMix_202311115.D
x10 6
10
57.1
49.0
62.0
31.1
42.0
Counts vs. Mass-to-Charge (m/z)

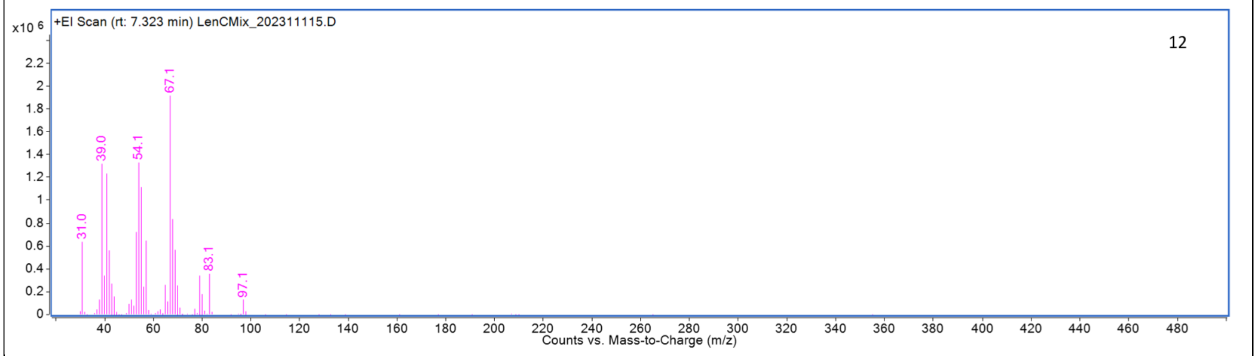
+EI Scan (rt: 7.323 min) LenCMix_202311115.D
x10 6
12
67.1
39.0
54.1
31.0
83.1
97.1
Counts vs. Mass-to-Charge (m/z)

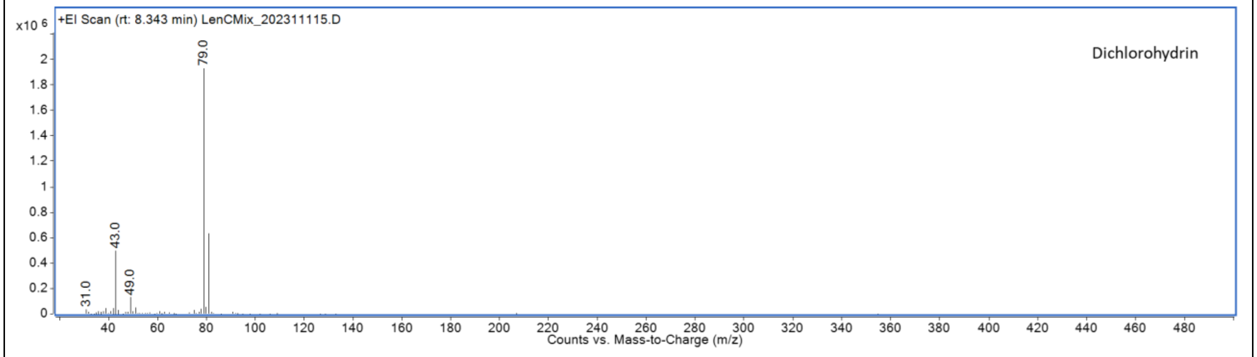
+EI Scan (rt: 8.343 min) LenCMix_202311115.D
x10 6
Dichlorohydrin
79.0
43.0
49.0
31.0
Counts vs. Mass-to-Charge (m/z)

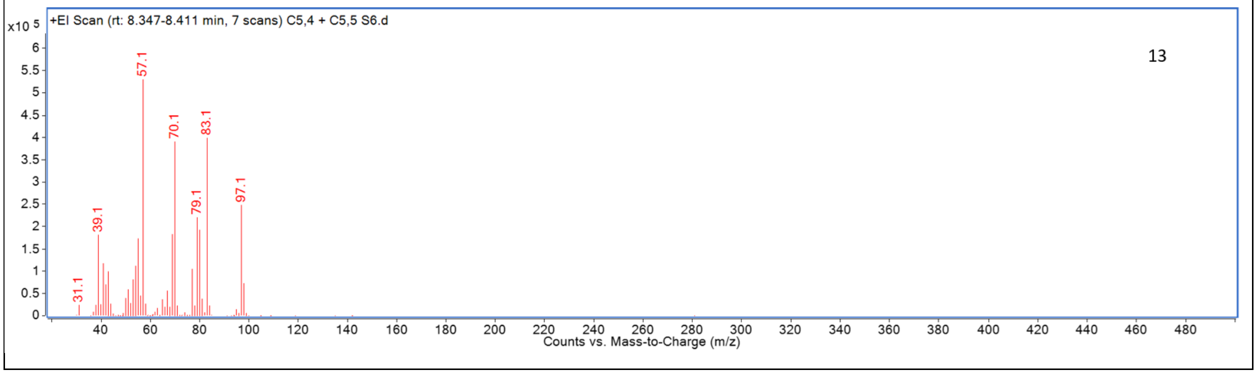
+EI Scan (rt: 8.347-8.411 min, 7 scans) C5,4 + C5,5 S6.d
x10 5
13
57.1
70.1
83.1
97.1
79.1
39.1
31.1
Counts vs. Mass-to-Charge (m/z)

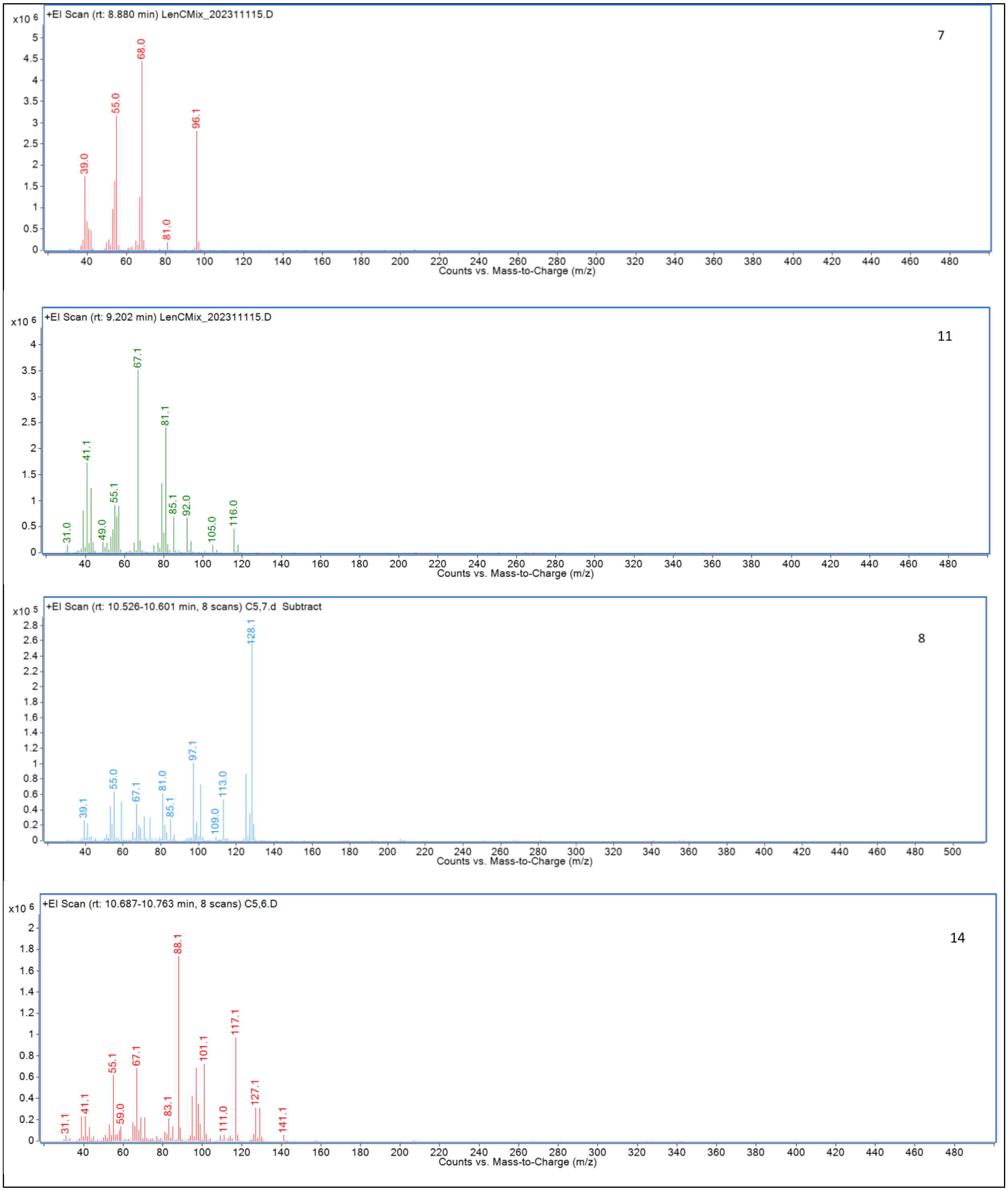

Quantitation of **12** was performed via GC-MS (Agilent 8890 GC-5977 MSD). The column used was an Agilent J&W HP-5ms GC Column, 30 m, 0.25 mm, 0.25 µm. The inlet was set to 250 °C. A split ratio of 50:1 was used with an injection volume of 1.0 µL. The column flow rate was 1.4

mL/min with helium as the carrier gas and an inlet pressure of 12.4 psi. The oven was initially set to 60 °C for 3 minutes, linearly ramped to 125 °C at 12 °C/min with no hold.

Structures & IDs:

Exact Mass: 98.07

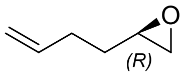

12

<u>Instrument Type:</u> Agilent 8890 gas chromatograph (GC) with a 5977 mass spectrometer detector (MSD)

<u>Conditions:</u>

<u>Column:</u> J&W HP-5ms GC Column, 30 m, 0.25 mm, 0.25 µm

<u>Inlet Pressure:</u> 12.4 psi | <u>Split Ratio:</u> 50 : 1 | <u>Split Flow:</u> 70 mL/min

<u>Column flow:</u> 1.4 mL/min | <u>Injection Temp:</u> 250 °C | <u>Injection volume:</u> 1 µL

<u>Total Flow:</u> 74.4 mL/min | <u>Solvent Delay:</u> 2 min | <u>Runtime:</u> 8.4 min

<u>Temperature Program:</u>

Time (min)	Ramp (°C/min)	Temp (°C)	Hold (min)
Initial	-	60	3
-	12	200	-

<u>MS Parameters:</u>

Transfer Line Temp (°C)	250
Source Temp (°C)	230
Quad Temp (°C)	150
Electron Energy (eV)	70
Mass Range	40-1000

<u>Sample preparation:</u> Prepare solutions at approximately 0.5 – 1 mg/mL in acetonitrile or methanol for quantitative analysis. **12** is quite volatile and must be prepared carefully for quantitation.

Retention Times

**Compound**	***m/z***	**Time (min)**
**12**	97, 67	2.75

Representative Chromatogram

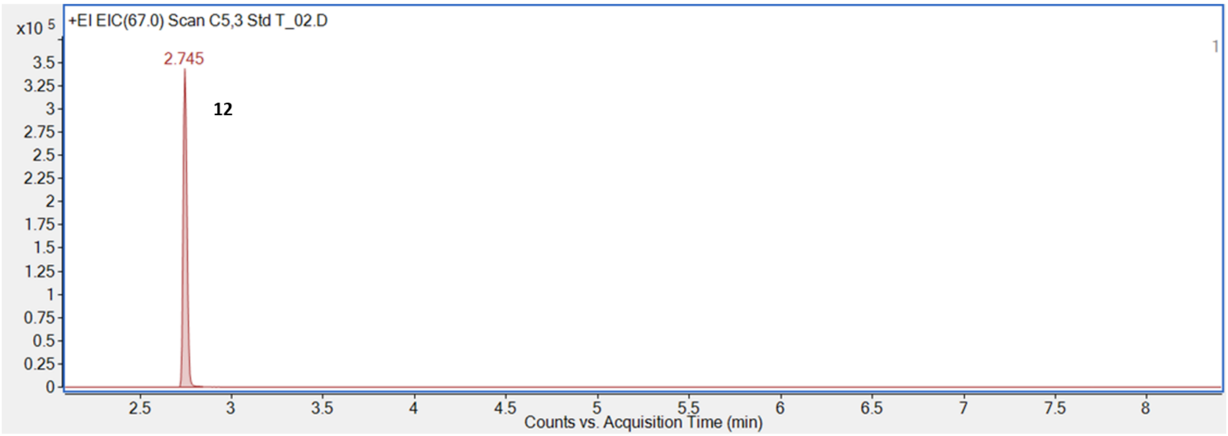

Mass spectra:

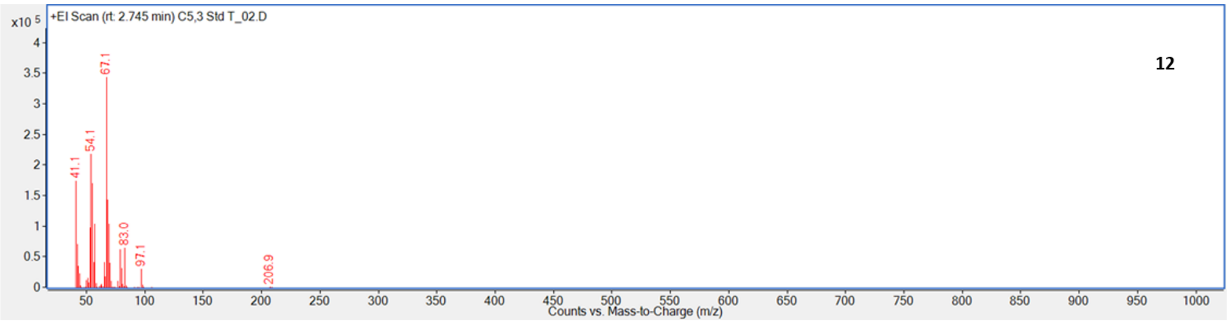

GC-FID Chiral Methods. Chiral purity of **12** was monitored via GC-MS (Agilent 6890 GC-FID). The column used was a Restek RT-GammaDEXsa (30 m x 0.25 mm x 0.25 µm). The inlet was set to 250 °C. A split ratio of 50:1 was used with an injection volume of 1.0 µL. The column flow rate was 1.4 mL/min with helium as the carrier gas and an inlet pressure of 16.4 psi. The oven was initially set to 60 °C and held for 16 minutes.

Structures & IDs:

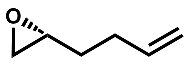

12 - (R) Enantiomer
Exact Mass: 98.07

Instrument Type: Agilent 6890 gas chromatograph (GC) with a flame ionization detector (FID)

Conditions:

Column: RT-GammaDEXsa (30 m x 0.25 mm x 0.25 µm)

Inlet Pressure: 16.4 psi | Split Ratio: 50 : 1 | Split Flow: 71.3 mL/min

Column flow: 1.4 mL/min | Inlet Temp: 250°C | Injection volume: 1 µL

Runtime: 16 min | H_2 flow: 35.0 mL/min | Air flow: 450 mL/min

Temperature Program: Isothermal at 60 °C | Makeup (N_2) Flow: 30.0 mL/min

FID Temperature: 250 °C

Sample preparation: Prepare solutions at approximately 5.0 mg/mL in methanol.

Retention Times

Compound	Time (min)
**12** (R) enantiomer	12.5
**12** (S) enantiomer	13.2

Notes: For accurate integration of **12**, draw through entire tail of the (R) peak and tangent skim the (S) peak.

Representative Chromatograms:

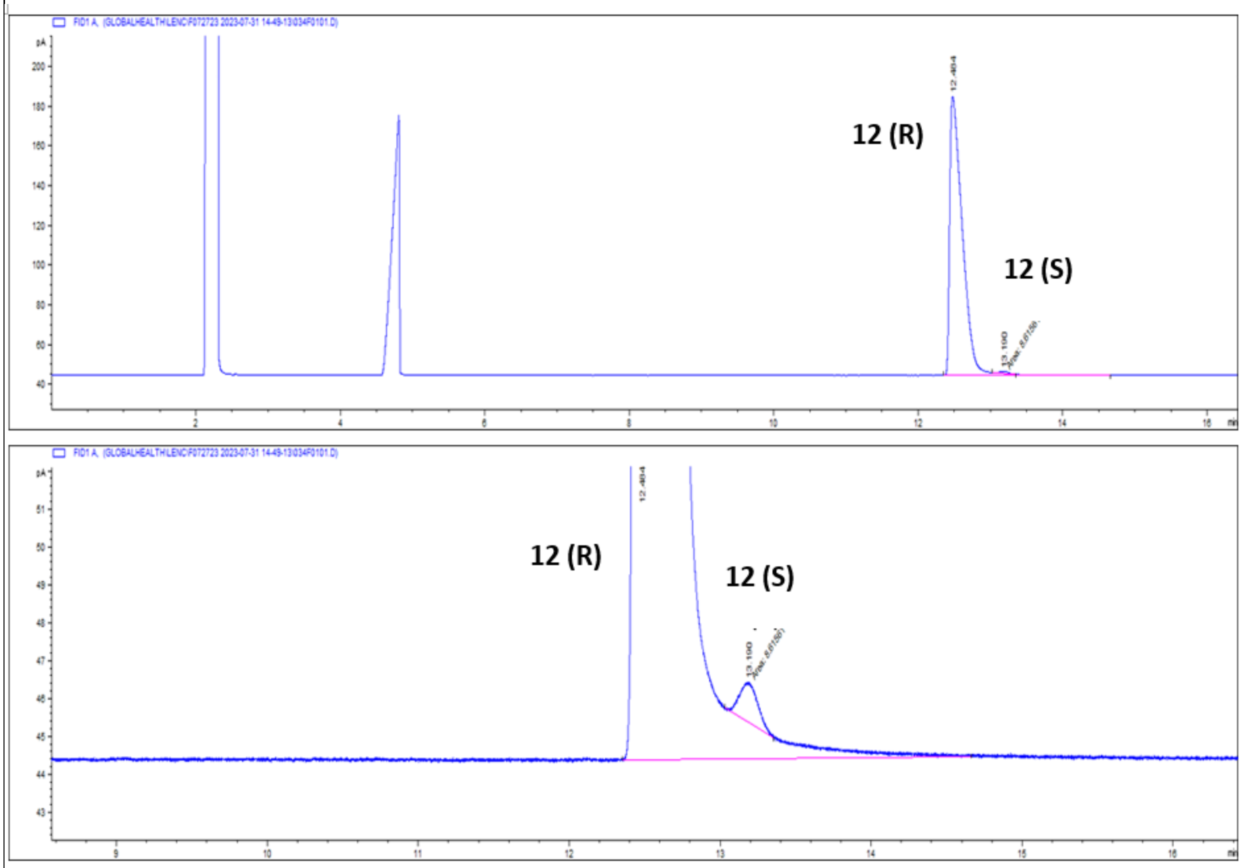

Chiral purity of **7** was monitored via GC-MS (Agilent 6890 GC-FID). The column used was a Restek RT-GammaDEXsa (30 m x 0.25 mm x 0.25 µm). The inlet was set to 200 °C. A split ratio of 50:1 was used with an injection volume of 1.0 µL. The column flow rate was 0.56 mL/min with helium as the carrier gas and an inlet pressure of 10.0 psi. The oven was initially set to 125 °C and held for 45 minutes.

Structures & IDs:

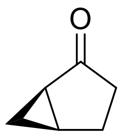

7
Exact Mass: 96.06

Instrument Type: Agilent 6890 gas chromatograph (GC) with a flame ionization detector (FID)

Conditions:

Column: RT-GammaDEXsa (30 m x 0.25 mm x 0.25 µm)

Inlet Pressure: 10.0 psi	Split Ratio: 50 : 1	Split Flow: 27.8 mL/min
Column flow: 0.56 mL/min	Inlet Temp: 200°C	Injection volume: 1 µL
Runtime: 45 min	H_2 flow: 35.0 mL/min	Air flow: 450 mL/min

Temperature Program: Isothermal at 125 °C   Makeup (N_2) Flow: 30.0 mL/min

FID Temperature: 200 °C

Sample preparation: Dilute samples in acetonitrile.

Retention Times

**Compound**	**Time (min)**
**7** Enantiomer 1	19.5
7 Enantiomer 2	21.0

Notes: Stereochemical assignments unknown

Representative Chromatogram:

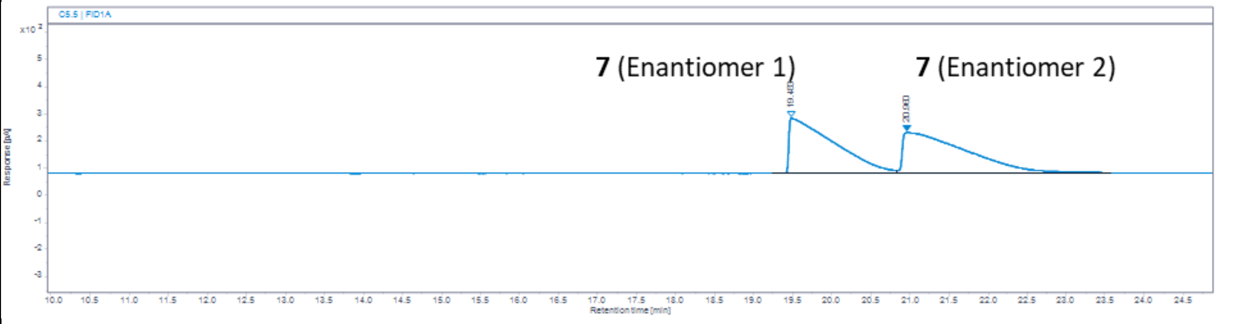

SFC Chiral Method. Chiral purity of **8** was monitored by SFC-DAD. This method utilized a ChiralPak IG-3 4.6 x 250 mm; 3 µm column. Flow rate was set to 2 mL/min with an injection volume of 5 µL. Column temperature was 25 °C. Isomers were separated isocratically with 95:5 CO2: methanol over 10 min and chromatograms were collected at 210 nm. Known enantiopure standards were not available for **8** and therefore peak assignments were not made.

Structures & IDs:

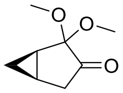

8
Exact Mass: 156.08

Instrument Type: Agilent 1260 super critical fluid chromatograph (SFC) with diode array detector (DAD)

Conditions:
Column: ChiralPak IG-3 4.6x250mm; 3 µm
Mobile Phase A: CO_2
Mobile Phase B: Methanol
Injection volume: 1 µL   Column temp: 25°C   Flow rate: 2.0 mL/min
BPR Pressure: 100 bar   BPR Temp: 60 °C
Detector wavelength(s): 210 nm
LC Gradient Table:

Time (min)	%A	%B
0.0	95	5
10.0	95	5

Sample preparation:
Prepare solution in methanol at approximately 5 mg/mL.

Retention Times

**Compound**	**Time (min)**
**8** Enantiomer 1	2.8
**8** Enantiomer 2	3.0

Notes: Stereochemical assignments unknown

Representative Chromatograms

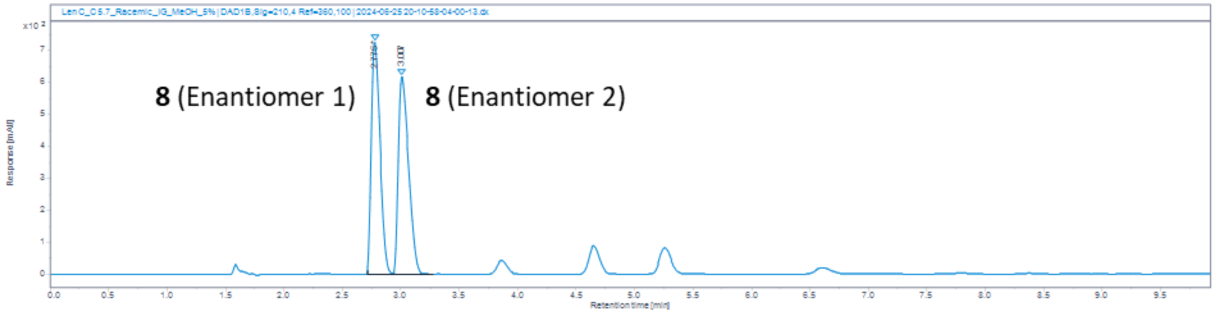

UV Spectra

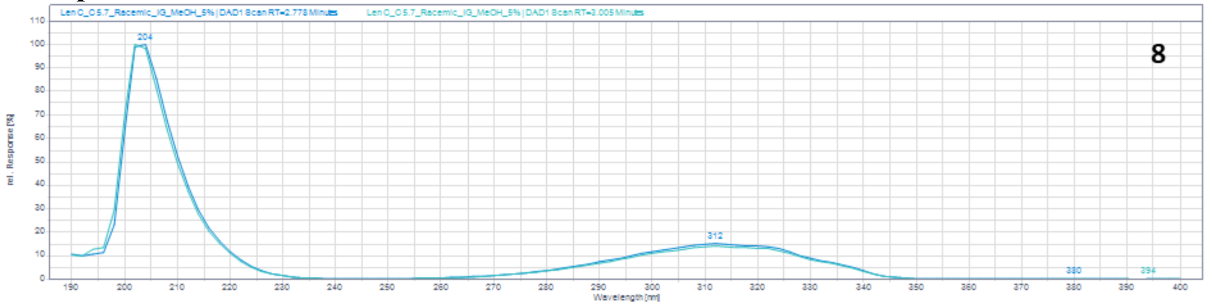

DRYING PROCESS DEVELOPMENT OF CRUDE SOLUTION OF 12

Two distinct methods were developed to dry the reaction mixture of **12**. The first approach was the use of molecular sieves (4Å) as drying reagent (Figure S1).

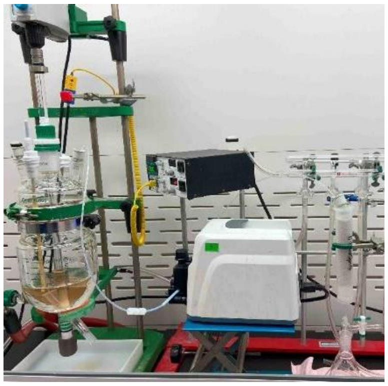

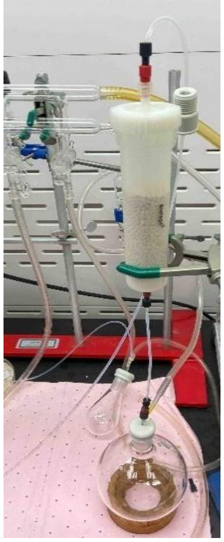

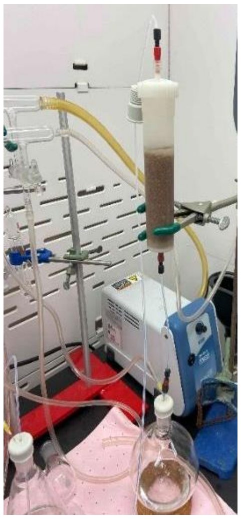

a). **First drying system**	**b). 2,3, and 4th drying**

Figure S1. Drying crude solution of 12 by passing through cartridge packed with molecular sieves (a, b)

The molecular sieves powder was first packed into a cartridge with screw connectors. The solution of **12** was pumped through the cartridge with a peristaltic pump. For example, The MTBE solution (reaction from 100g of (*R*)-epichlorohydrin according to the procedure as shown below, KF: 1.6%) was passed through a 100g molecular sieve cartridge (Biotage column). It is worth noting that the mass of molecular sieves was not optimized for drying. As shown in Table S1, the water content decreased to 0.55% after the first cycle of drying. The overall mass of the solution dropped to 454g while most of the compound **12** passed through the cartridge. Notably, the molecular sieves absorbed not only water but also some impurities as the purity of **12** was improved after the first drying. Pumping the dried solution of **12** through the same cartridge a second time, the resultant solution of **12** showed a KF of 0.04% which is dry enough to perform well in the next Hodgson cyclopropanation. To further evaluate the efficiency of the drying process, third and fourth drying cycles were carried out, and the water content improved to 0.02% after the third time drying but there was no improvement during the fourth drying. The total mass loss during this drying was only approximately 2%. The mass loss of compound **12** was 3% (3g in total) after four drying cycles and the purity of **12** increased to 98.5 A% (GCMS-TIC). The two-step overall yield was boosted to 83% after this drying process.

Table S1. Drying crude solution of 12 by passing through a cartridge packed with molecular sieves powder

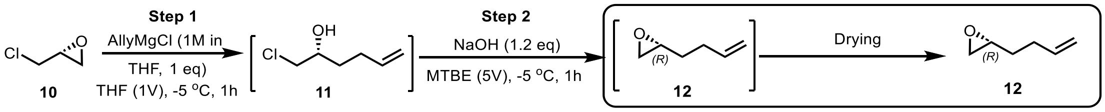

**12** in MTBE[1]	Crude before drying[2]	Numbers of drying cycles			
		1st	2nd	3rd	4th
Total mass of crude (g)	523g	454	-	-	446
KF (%) of crude	1.6	0.55	0.04	0.02	0.02
Mass of water content	7.3g	2.5g	-	-	0.09g

**12** in solution wt%[3]	17.6	20.0	-	-	20
**12** mass by wt%	92g	90g	-	-	89g
**12** A% (GCMS-TIC)	90[4]	93[5]	-	-	98.5%[6]

[1]After treatment with aq NaOH, the telescoped reaction mixture from **10** (100g) was washed with water, and brine before drying. [2]Cartridge packed with molecular sieves powder (100g, 4Å), the reaction crude was pumped through the cartridge by a peristaltic pump at 25 °C under a rate of 20mL/min [3]Wt% was obtained by thin-film GC-MS method with comparison to a known standard. [4]MTBE and THF solvent peaks were excluded, containing 1.5% of epichlorohydrin and 8% of **11**. [5]MTBE and THF solvent peaks were excluded, containing 1.5% of epichlorohydrin and 5.4% of **11**. [6]MTBE and THF solvent peaks were excluded, containing 1.5% of epichlorohydrin.

The second approach was azeotropic distillation, which is more practical in large-scale synthesis (See below in experimental section). To initiate the study of azeotropic distillation, a crude solution of **12** was prepared from the reaction of 148g of (*R*)-epichlorohydrin (**10**) with allylMgCl, followed by NaOH treatment (770g crude in total). The water content of this crude solution before drying was 1.45% by KF. The in-solution purity of **12** was 98.8 A% (GCMS-TIC) (Table S2). It is known that MTBE forms an azeotropic mixture with water (b.p. 53 °C), and the water content of this binary azeotropes was 4 wt%. Thus, the crude solution of **12** was heated at 70 °C under atmospheric pressure with a Dean-Stark trap for 12 h and approximately 10g of water was removed. The resulting solution was sufficiently dry for the next step. Notably, the mass loss of **12** was 4% in this azeotropic distillation process and the two-step overall yield was 87% after drying of the reaction mixture (Table S2). Compared to the drying process with molecular sieves, the azeotropic distillation is operationally simple and scalable. As a result, the latter drying approach was used to dry the crude **12** in our telescoping process development to synthesize **7**.

Table S2. Drying the crude solution of 12 by azeotropic distillation with Dean-Stark trap at 70 °C

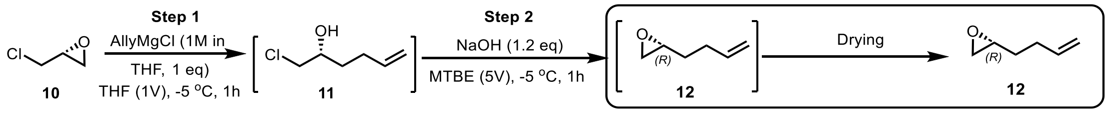

**12** in MTBE	Before azeotropic distillation[1]	After azeotropic distillation
Total mass of crude (g)	807	776
KF (%) of crude	1.4	0.19
Mass of water content	11.3g	1.5g

**12** in solution wt%[2]	19%	17.6%
**12** mass by wt%	152g	137 g
**12** A% (GCMS-TIC)	98.9%[3]	98.8%[4]

[1]After treatment with aq NaOH, the telescoped reaction mixture from **10** (148g) was washed by water, and brine before azeotropic distillation. [2]Wt% was obtained by qNMR with comparison to a known standard (mesitylene). [3]MTBE and THF solvent peaks were excluded, containing epichlorohydrin 1.1%. [4]MTBE and THF solvent peaks were excluded, containing epichlorohydrin 1.2%; the higher percentage of epichlorohydrin after azeotropic distillation might be probably due to a small amount of loss of **12** during the distillation.

REDUCTION OF THE OPERATING SOLVENT VOLUMES IN HYDROXYLATION

A total of 37V of solvents was required in this I_2-based hydroxylation (Table S3, Entry 1). Therefore, an investigation to lower the solvent volume was conducted. The initial effort was to utilize NaOMe (25 wt%, 6V) without diluting the mixture (Table S3, Entry 2). Under the standard charging sequence, the reaction of **7**, NaOMe/MeOH (6V), and I_2/MeOH (18V) yielded a smaller solvent volume to 24V, however, **14** was formed in a slightly lower purity profile (69 A% GCMS-TIC). As noted, the addition of I_2/MeOH to a mixture of **7**/NaOMe/MeOH became exothermic, and so the slow addition of a solution of I_2/MeOH (18V) is critical to afford a clean profile of **14**. It is assumed that a slow addition of **7** to the mixture of I_2/NaOMe/MeOH might also mitigate the exothermic heat generation thus allowing to reduce the amount of MeOH to make a solution of I_2. Encouragingly, the new process, with a slow addition of **7** to a mixture of I_2 and NaOMe, proceeded smoothly to produce **14** (Table S3, Entries 3-5). The reaction of I_2/MeOH (18V), NaOMe (6V) and **7** afforded **14** in good yield (75 A% GCMS-TIC) (Table S3, Entry 3). The same process with I_2/MeOH (10V) produced 79 A% GCMS-TIC of **14**, reducing the total solvent volume to 16V (Table S3, Entry 4). Additionally, the solvent volume was further decreased by using solid I_2 and NaOMe (6V), without any further dilution with methanol (Table S3, Entry 5). It is noted that the reaction with the charging alternative sequence of **7**, $I_{2,}$ and NaOMe resulted in a low purity profile of **14** (Table S3, Entries 6-7). Ultimately, the reaction of NaOMe (6V), solid I_2, and **7** in order afforded **14** in excellent yield and this hydroxylation process dramatically reduced the total solvent volume to 6V, which is suitable for further scale-up.

Table S3: Investigation of an addition order of reagents to reduce the solvent volumes

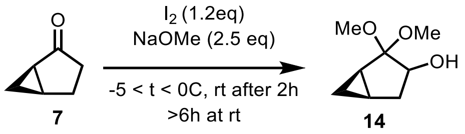

Entry[1]	Charging order			Total solvent (V)	**14** (IPC, A%, GCMS-TIC)[2]
	1st	2nd	3rd		
1	**7** (neat)	NaOMe / MeOH (19V)	I_2 / MeOH (18V)	37	87
2	**7** (neat)	NaOMe (6V)	I_2 / MeOH (18V)	24	69
3	I_2 / MeOH (18V)	NaOMe (6V)	**7** (neat)	24	75
4	I_2 / MeOH (10V)	NaOMe (6V)	**7** (neat)	16	79
5	NaOMe (6V)	I_2(s)	**7** (neat)	6	87
6	I_2 / MeOH (10V)	**7** (neat)	NaOMe (6V)	16	50
7	I_2 (s)	**7** / MeOH (6V)	NaOMe (6V)	12	40

[1]Typical procedure: NaOMe (2.4 eq) in MeOH, **7** (1 g), I_2 (1.2 eq), MeOH at -5 to 0 °C according to the addition order and conditions as shown in the table. [2]A% of IPC was measured by GCMS-TIC.

EXPERIMENTAL PROCEDURE

Typical procedure of preparation of 12 for drying of the mixture in telescoped process development (based on the entry 2 in Table 1)

1. ***Synthesis of (R)-1-chlorohex-5-en-2-ol (11)***

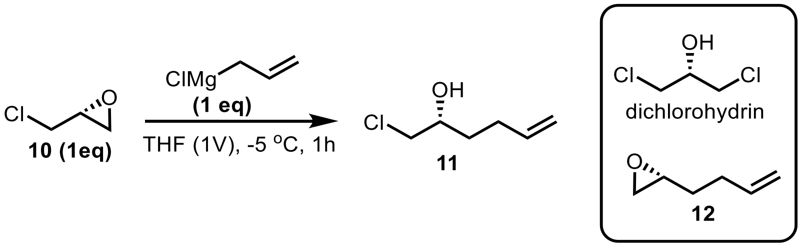

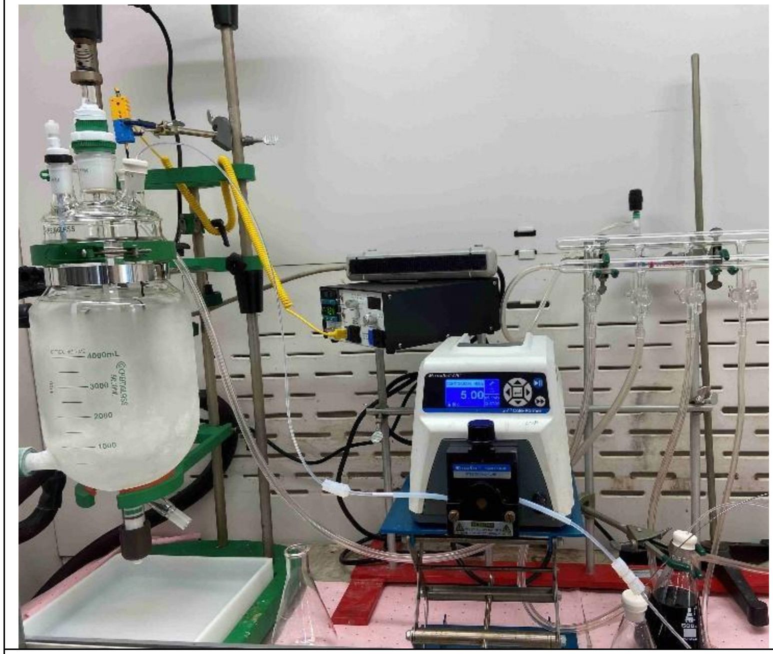	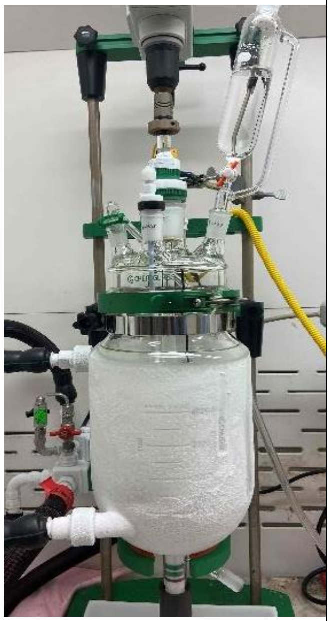
**(Grignard addition)**	**(MeOH and HCl quenching)**

THF (150mL, 1V) and (*R*)-epichlorohydrin **10** (148g, 1.6mol, 1eq) were charged to a 5L ChemRxnHub reactor under a nitrogen atmosphere. This mixture was cooled at -25°C (internal temperature was -14.5°C) using a Huber circulating chiller. When the internal temperature achieved -14.5°C, allylmagnesium chloride (800mL, 1.6mol, 1eq, 2M in THF) was added using a peristaltic pump at a flow rate of 5 mL/min, maintaining the internal temperature below -5.0°C. After addition, this mixture was stirred at the same temperature for an additional 1h. After completion of the reaction (monitored by GC-MS), methanol (162mL, 4.0mol, 2.5eq) was added dropwise, keeping the internal temperature below 0°C, followed by addition of HCl (1.6L, 2M, 2.0eq) at 0°C. The circulating cooling system was turned off and MTBE (740mL) was added. The organic layer was collected and washed with HCl (300mL, 2M) and water (300mL), respectively. This resulting organic layer (1360mL corresponding to 1150g) gave an in-solution yield of 99% (corrected weight 215.3g) **11** assayed by GC-MS (19 wt%), with 95 A% GCMS-TIC (excluding solvent peaks etc.), ee: 99.9% and containing 0.7 A% of dichlorohydrin and 1.8 A% of epoxide **12** by GCMS-TIC. The crude compound **11** was used in the next step without further purification. A small amount of concentrated solution was used for ^{1}H NMR analysis.

^{1}H NMR (600 MHz, $CDCl_3$) δ 5.78 (ddt, *J* = 17.0, 10.2, 6.7 Hz, 1H), 5.01 (ddd, *J* = 17.1, 3.4, 1.7 Hz, 1H), 4.94 (ddd, *J* = 10.2, 3.0, 1.3 Hz, 1H), 3.55 (dd, *J* = 11.1, 3.9 Hz, 1H), 3.44 (dd, *J* = 11.1, 6.7 Hz, 1H), 3.05 (d, *J* = 5.7 Hz, 1H), 2.31 (s, 2H), 2.25 – 2.08 (m, 2H), 1.63 – 1.54 (m, 2H).

MS-EI (*m/z*) ($M^{\cdot+}$): 134.

2. ***Synthesis of R-(+)-1,2-epoxy-5-hexene (12)***

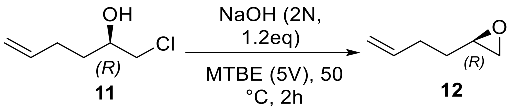

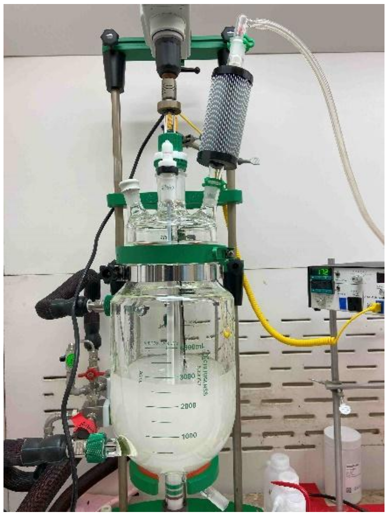

Reflux

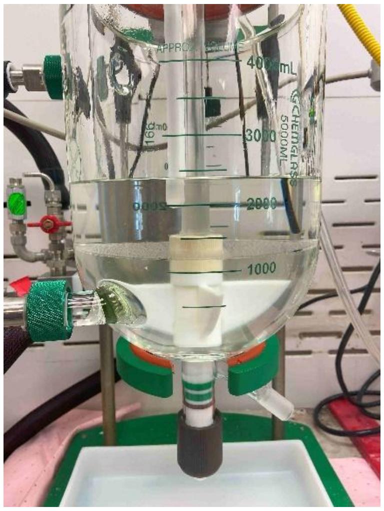

Extraction

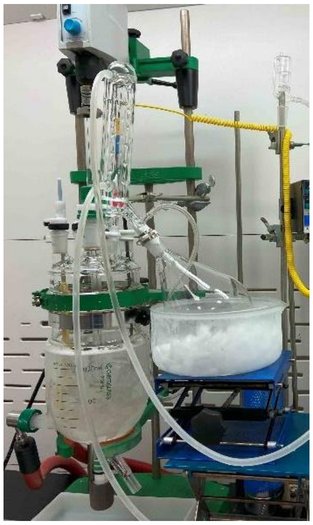

Concentration

A solution of chlorohydrin **11** in MTBE (1150.47g, 19 wt%, ~1.4L) was charged in a 5L ChemRxnHub reactor, then an aqueous solution of NaOH (960mL, 1.2eq, 2N) was added. The mixture was heated to 50°C and stirred for 2h. After completion of the reaction, the organic layer was collected and washed with water (250mL × 4) until the aqueous layer had pH = 7. The organic layer (~1.0L, 807g, 19 wt% by qNMR, KF = 1.4%, 98.9A% by GCMS) was then used for drying (Figure S2).

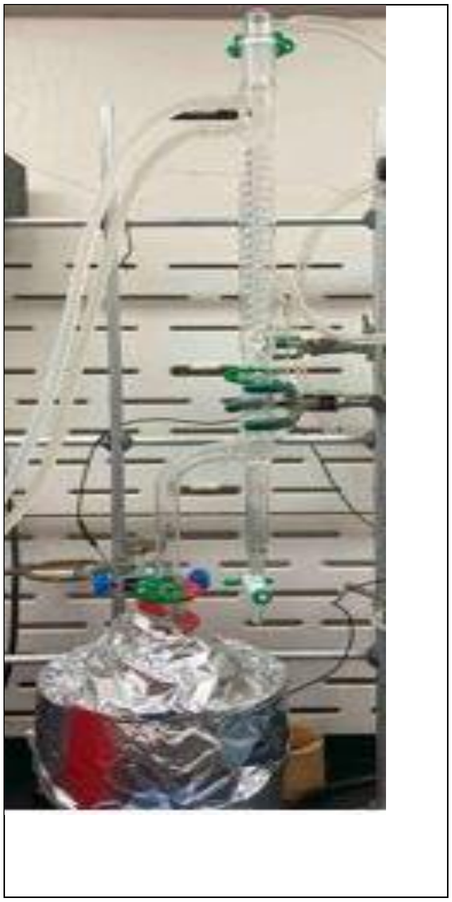

Figure S2. Drying crude solution 12 by azeotropic distillation with Dean-Stark trap

The above MTBE solution (~1.0L, 807g, KF: 1.4%) was charged to a 2L RBF with a Dean-Stark trap. The solution was refluxed at 70°C for 16h and about 10g of water was removed. The KF of the resulting MTBE solution was reached to 0.19%. This resulting organic layer (776g) gave an in-solution yield of 87% (corrected weight 136g, over two steps from **10**) epoxide **12** assayed by GC-MS (17.6 wt% purity by qNMR), with 98.8 A% GCMS-TIC (excluding solvent peaks etc.) and contained 1.2 A% (GCMS-TIC) of epichlorohydrin. The crude compound **12** was used in the next step without further purification. A small amount of concentrated solution was used for ^{1}H NMR analysis.

^{1}H NMR (600 MHz, $CDCl_3$) δ 5.81 – 5.72 (m, 1H), 5.02 – 4.87 (m, 2H), 2.88 – 2.81 (m, 1H), 2.67 (t, *J* = 4.5 Hz, 1H), 2.40 (dd, *J* = 5.0, 2.7 Hz, 1H), 2.20 – 2.07 (m, 2H), 1.61 – 1.48 (m, 2H).
MS-EI (*m/z*) ($M^{\cdot+}$): 98

Typical procedure of synthesis of (*1S,2R,5R*)-bicyclo[3.1.0]hexan-2-ol (13)

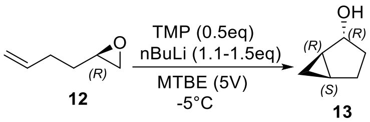

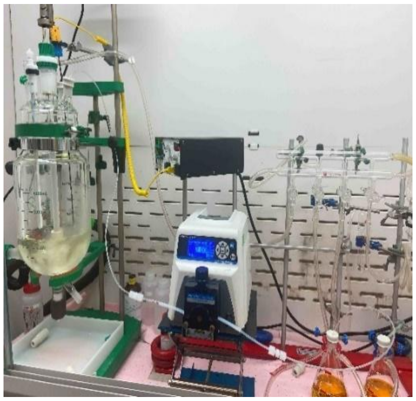

Reaction setup

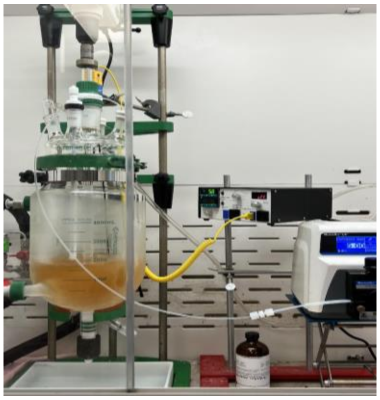

***nBuLi* addition**

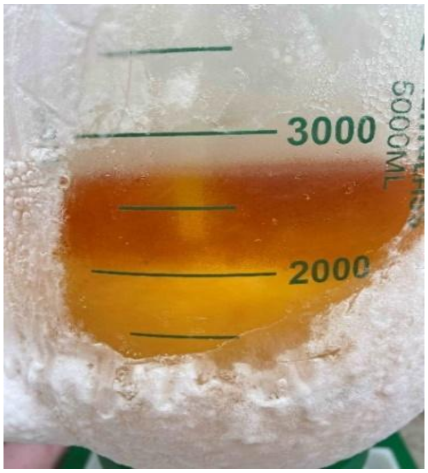

Quenching with 3.0 M HCl

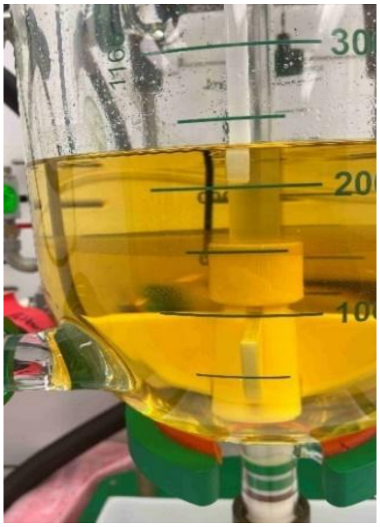

Upon warming

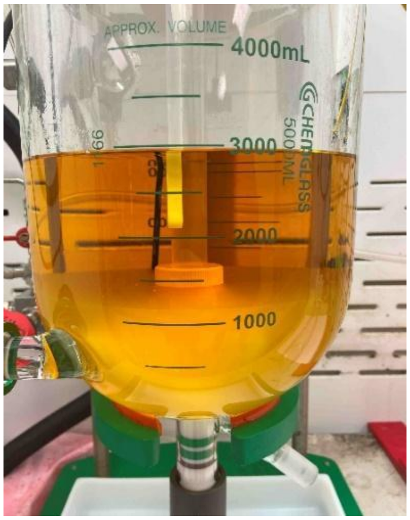

MTBE extraction

A 5L ChemRxnHub reactor was charged with a dried solution of epoxide **12** in MTBE (776g, 17.6wt%). TMP (118mL, 0.5eq) was added to the reactor. This mixture was cooled at -10°C (internal temperature was -9.9°C) using a Huber circulating chiller. When the internal temperature achieved -9.9°C, n-butyllithium (612mL, 1.5mol, 1.1eq, 2.5M in hexane) was added using a peristaltic pump with a flow rate of 4.0 mL/min, maintaining the internal temperature below -

5.0°C. After the addition, this mixture was stirred at the same temperature for an additional 1h. After completion of the reaction, HCl (745mL, 3M, 1.6eq) was added dropwise, keeping the internal temperature below 25°C. The circulating cooling system was turned off, and the aqueous layer was drained (pH = 10). Then, more HCl (235mL, 3M, 0.5eq) was added to wash the organic layer (pH = 1). The combined aqueous layer was charged back to the reactor and extracted twice with 5V and 2.5V of MTBE (685mL and 340mL). This combined organic layer (2640mL) was concentrated at 40°C under vacuum (200 – 340mbar) to about 5V. This resulting organic layer (699.09g, 900mL) afforded bicycloalcohol **13** with an in-solution yield of 72% (corrected weight 98.08g) by qNMR (14.03 wt%, 89.2 A% (excluding solvent peaks etc.) or 11.6% purity by GC-MS). The crude compound **13** was used in the next step without further purification.

Purification by distillation: the desired compound **13** can also be purified by distillation at this stage. The crude **13** was distilled under 455 to 310 mbar vacuum at 70°C to remove MTBE and further distillation at 45°C and 1-6 mbar to afford the desired compound **13** as an oil with 65-70% isolated yield.

^{1}H NMR (600 MHz, $CDCl_3$) δ 4.21 (d, J = 4.7 Hz, 1H), 1.97 – 1.72 (m, 2H), 1.65 (dd, J = 12.3, 8.3 Hz, 1H), 1.53 (dd, J = 14.5, 8.3 Hz, 1H), 1.44 – 1.37 (m, 1H), 1.31 (ddd, J = 12.4, 8.2, 4.8 Hz, 2H), 0.41 (dd, J = 7.7, 5.7 Hz, 1H), 0.00 (dd, J = 7.9, 4.2 Hz, 1H).

^{13}C{^{1}H} NMR (151 MHz, $CDCl_3$) δ 74.5, 30.4, 26.9, 24.5, 16.1, 6.9.

IR (ATR) ν_{max} = 3328.5, 2935.3, 2870.1, 1328.8, 1177.8, 1101.4, 1043.7, 984.0, 870.3, 808.8, 717.5 cm^{-1}.

MS-EI (*m/z*) ($M^{\cdot+}$): 98

$[\alpha]_D^{20}$ (deg·mL·g^{-1}·dm^{-1}) (MeOH (10mg/mL) at 20 °C under 589nm): +23.96

Typical procedure of synthesis of (*1S,5R*)-bicyclo[3.1.0]hexan-2-one (7)

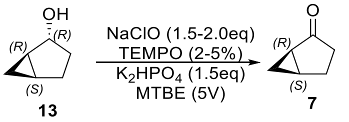

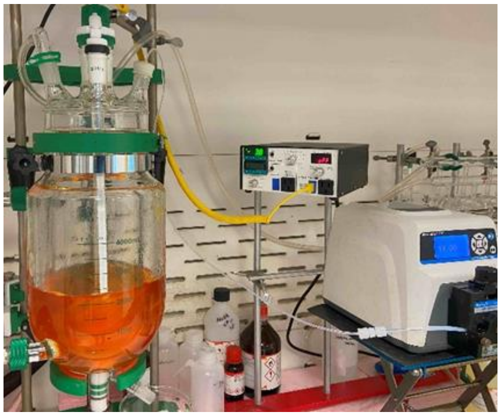

Bleach oxidation setup

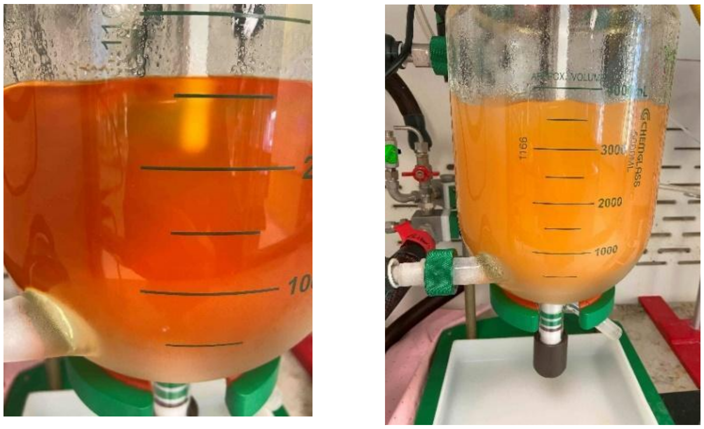

During the bleach addition

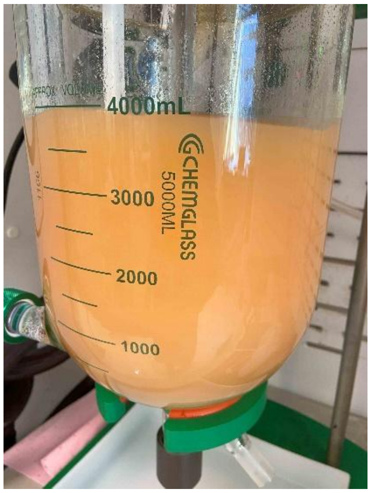

After bleach addition

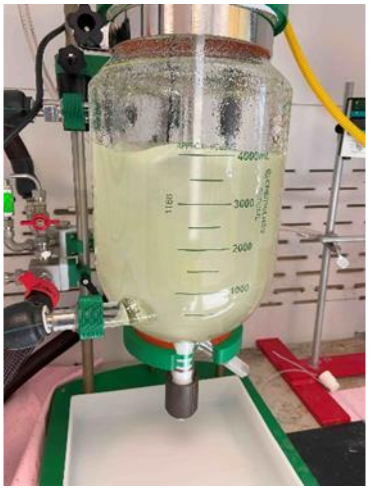

After completion of the reaction

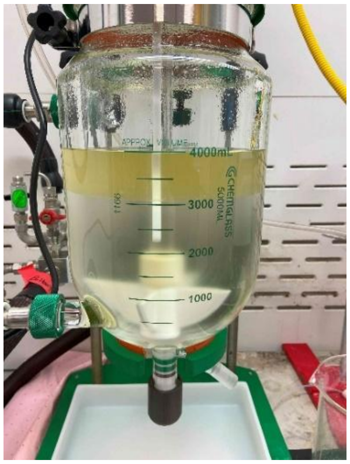

Extraction with MTBE

A 5L ChemRxnHub reactor was charged with a solution of bicycloalcohol **13** in MTBE (699.09g, 14.03wt%). To the reactor was added a solution of K_2HPO_4 (366.3g, 2.1 mol, 1.5 eq) in water (555 mL, 22 eq) followed by TEMPO (5.44g, 34.6 mmol, 0.025 eq). This mixture was cooled at -5°C (internal temperature was -3.8°C) using a Huber circulating chiller. When the internal temperature achieved -3.8°C, 10% sodium hypochlorite solution (1,3L, 2.1mol, 1.5eq) was added using a peristaltic pump with a flow rate of 12 mL/min, maintaining the internal temperature below 5.0°C. After the addition, the circulating cooling system was turned off, and when the internal temperature achieved 20°C, the reaction was stirred for an additional 3h. After completion of the reaction,

sodium sulfite solution (61.1g, 0.35 eq in 280 mL of water) was added, keeping the internal temperature below 30°C. The organic layer was separated and the aqueous layer was extracted twice with 5V and 2.5V MTBE (685mL and 340 mL). This combined organic layer (1840 mL, 87 A%) was concentrated at 40 °C under vacuum (200 – 340 mbar) to remove MTBE. This resulting residue 196.33g (210mL) gave an in-solution yield of 94.1% (90.4g) bicyclo ketone **7** assayed by qNMR (46.06 wt%).

Purification: The resulting crude material was distilled at 25-40°C under vacuum (4 – 1.8 Torr) with a vapor temperature between 30 – 40°C to afford the desired bicyclo ketone **7** (91.55g, 4-step overall yield: 55% based on the purity, purity: 92 A% by GCMS-TIC and 94 wt% by qNMR, *ee*: 100%).

^{1}H NMR (600 MHz, $CDCl_3$) δ 2.19 – 1.94 (m, 5H), 1.78 – 1.70 (m, 1H), 1.22 – 1.14 (m, 1H), 0.96 – 0.88 (m, 1H).
^{13}C{^{1}H} NMR (151 MHz, $CDCl_3$) δ 215.2, 31.4, 27.4, 22.6, 21.6, 13.5.
MS-DART(*m/z*) (MH^+): 97.1.
IR (ATR) ν_{max} = 3328.5, 2935.3, 2870.1, 1328.8, 1177.8, 1101.4, 1043.7, 984.0, 870.3, 808.8, 717.5 cm^{-1}.
GC (RT-GammaDEXsa (30 m × 0.25 mm × 0.25 μm), column flow = 0.56 mL/min, H_2 flow = 35.0 mL/min, air flow = 450 mL/min) t_R = 19.1 min (100%), 21.0 min (0%).
Specific rotation: $[\alpha]_D^{20}$ = +20.79 (deg·mL·g^{-1}·dm^{-1}) (measured in MeOH (10mg/mL) at 20 °C under 589nm)

Typical procedure of synthesis of (1R,5R)-2,2-dimethoxybicyclo[3.1.0]hexan-3-ol (14)

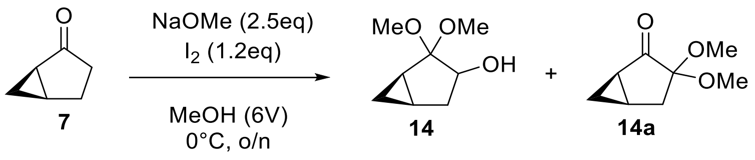

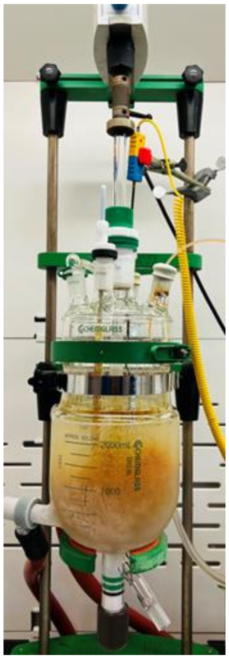	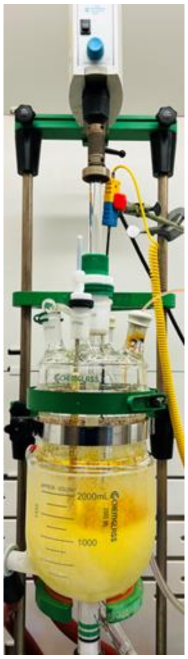	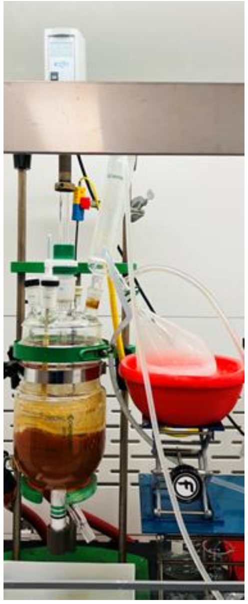	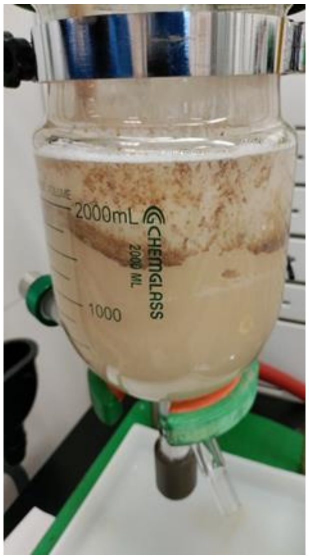
**7 + NaOMe + I_2**	**Color change during the reaction**	**Distillation of MeOH**	**After charging DCM**
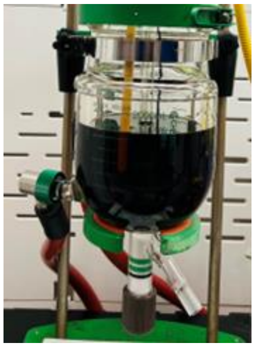	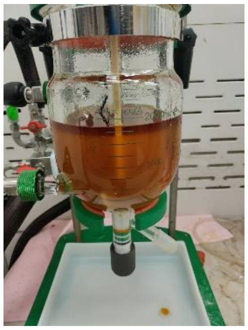	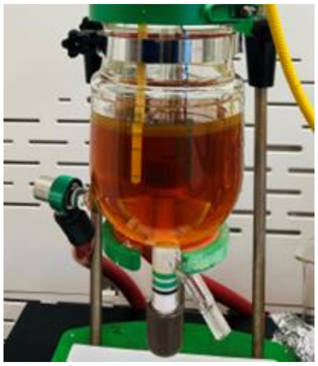	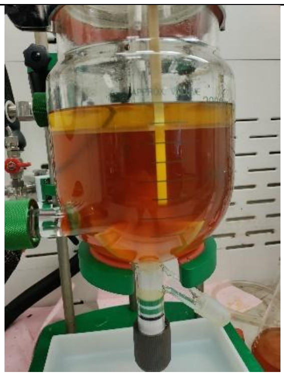
**Water wash**	**aq. Na_2SO_3 wash**	**1st Brine wash**	**2nd Brine wash**

A 2L ChemRxnHub reactor was charged MeONa/MeOH (563.3 g, 596 mL, 25 wt%, 2.52 eq, 2.607 mol) and cooled to -10°C under N_2. Iodine (315.0 g, 1.2 eq, 1.241 mol) was added to the reactor once the internal temperature reached -5°C. A slight exotherm was noticed (2 to 10°C) during this operation. The resulting solution was allowed to stir for 15 to 20 min at -5°C to -10°C. Then, (*1R,5S*)-bicyclo[3.1.0]hexan-2-one **7** (105.80 g, 94 wt%, 1 eq, 978 mmol) was added dropwise using a peristaltic pump (2 mL/min) maintaining NMT 0 °C internal temperature. The reaction mixture was stirred at 0 °C overnight. After completion of the reaction (monitored by GCMS and ^{1}H NMR), the reaction mixture was concentrated under vacuum (45°C) to remove the

MeOH. The resulting residue was dissolved in DCM (1000 mL, 10V) and stirred for 10 min. Water (200 mL, 2 V) was added, and the mixture was stirred for another 10 min. The DCM layer was separated, and the aqueous layer was extracted with DCM (300 mL × 1, 3V). The combined DCM solution was charged to the reactor and washed with saturated Na_2SO_3 solution (200 mL × 2, 2 V) and brine (200 mL, 2 V). The DCM layer was collected (**14**: 91 A%; **14a**: 6 A%; **7**: <1 A% by GCMS-TIC) and concentrated *in vacuo* to afford the crude product **14** (160.35 g, 82% qNMR purity, 80% yield; 2 A% byproduct **14a**) as a pale-yellow oil. The crude product was used for the next step out with further purification.

Purification by distillation: the desired compound **14** can also be purified by distillation at this stage. The crude **14** was distilled at 40°C under 1 mbar to afford the desired compound **14** in 70-75% isolated yield.

^{1}H NMR (600 MHz, $CDCl_3$) δ 3.97 (d, *J* = 7.1 Hz, 1H), 3.44 (s, 3H), 3.25 (s, 3H), 2.37 (s, 1H), 2.24 – 2.15 (m, 1H), 1.78 (dd, *J* = 14.1, 0.8 Hz, 1H), 1.53 – 1.44 (m, 1H), 1.38 (dd, *J* = 8.8, 5.0 Hz, 1H), 0.82 (dd, *J* = 9.0, 4.2 Hz, 1H), 0.58 (ddt, *J* = 8.5, 5.0, 1.2 Hz, 1H).

^{13}C{^{1}H} NMR (151 MHz, $CDCl_3$) δ 110.3, 72.3, 51.3, 49.2, 34.1, 21.8, 14.9, 8.5.

IR (ATR) ν_{max} = 3475.7, 2942.7, 2834.6, 1448.1, 1366.1, 1341.8, 1142.4, 1049.2, 1030.6, 978.4, 911.3, 814.4 cm^{-1}.

HRMS (ESI) *m/z*: calcd for $C_8H_{14}O_3 \cdot Na^+$ = $[M+Na]^+$ 181.0841, found181.0844

Typical procedure of Synthesis of (1R,5R)-2,2-dimethoxybicyclo[3.1.0]hexan-3-one (8)

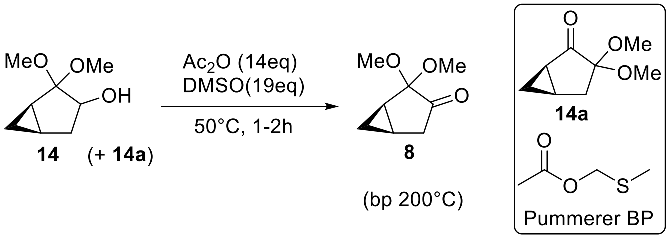

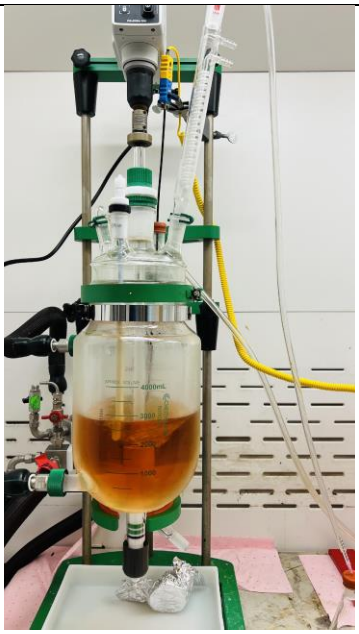	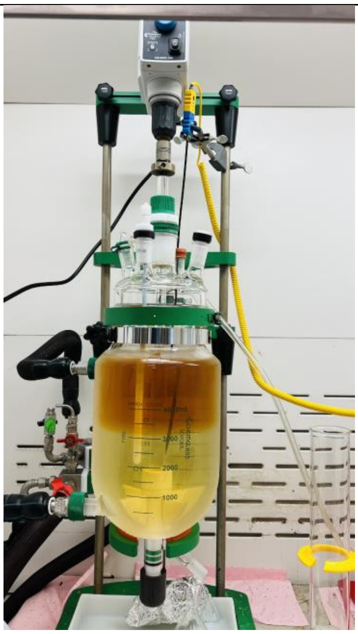	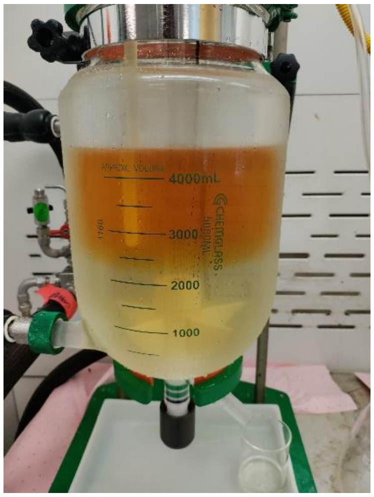
**Reaction mixture**	**Work-up**	**Work-up**

A 5L ChemRxnHub reactor was equipped with a thermocouple, baffle, N_2 flow and a bleach trap. DMSO (1.2 kg, 1.1L, 19 eq, 15.8 mol) and (*1R,5R*)-2,2-dimethoxybicyclo[3.1.0]hexan-3-ol (*1R,5R*)-2,2-dimethoxybicyclo[3.1.0]hexan-3-ol **14** (160.3 g, 832 mmol, 82% purity) were charged to the reactor at 25 °C. Acetic anhydride (1.2 kg, 1.1L, 14 eq, 11.6 mol) was added at the same temperature. The reaction mixture was heated to 50 °C and stirred for 1.5 h under N_2. After completion of the reaction (once **14** was <5 A% by GC-MS and ^{1}H NMR), the reaction mixture was cooled to 0°C and diluted with water (1.6L, 10V) and EtOAc (1.6L, 10V). The mixture was stirred at 0°C for 30 min. The water layer was separated and extracted with EtOAc (800mL × 2). The combined EtOAc layer was washed with sat. $NaHCO_3$ (1000 mL × 3) to pH = 6-7 and then brine (1L). The resulting EtOAc layer (**8**: 80 A%; **14a**: 5 A%; **BP**: 15 A% by GCMS-TIC) was concentrated in the reactor under 300 -50 torr at 50°C to afford the crude **8** as a pale-yellow liquid (189 g, 40 wt% by GC).

The crude product was further purified by vacuum distillation using a 12” Vigreaux column and a JKEM vacuum controller. The first fraction was collected at 50 °C under 35-7 torr, to purge a mixture of EtOAc, acetic anhydride, and Pummerer BP as the first fraction (69g). The resulting residue was distilled at 60 °C under 3 torr to afford **BP** as the major fraction (5.2 g). The resulting residue was distilled at 60 °C under 1-2 torr to afford **8** (77 g, two-step overall yield: 45%, 94 wt%

by qNMR, 95 A% by GCMS-TIC, containing < 1 A% the Pummerer **BP** and 3-4 A% **14a** by GCMS-TIC). The obtained **8** was dissolved in hexanes (390mL, 5V) and cooled to -40 to -45°C with overhead stirring and **8** was formed as an amorphous solid. Once precipitation of **8** was complete, the supernatant was suctioned through a 20 um glass inlet filter via a peristaltic pump at 20 mL/min until dry. The residue was quantitatively collected with MTBE and concentrated *in vacuo* to yield **8** as a pale-yellow oil (68 g, 98 wt% by qNMR, 97 wt% by GC, 100 A% by GCMS-TIC, two-step overall yield after precipitation: 41%).

^{1}H NMR (600 MHz, $CDCl_3$) δ 3.19 (s, 3H), 3.01 (s, 3H), 2.54 (ddd, *J* = 18.8, 5.5, 2.2 Hz, 1H), 2.04 (d, *J* = 18.7 Hz, 1H), 1.49 (td, *J* = 7.9, 4.2 Hz, 1H), 1.42 – 1.34 (m, 1H), 0.71 (dq, *J* = 8.2, 2.1 Hz, 1H), -0.01 (dt, *J* = 6.3, 4.3 Hz, 1H).
^{13}C{^{1}H} NMR (151 MHz, $CDCl_3$) δ 207.7, 102.9, 50.9, 50.5, 38.9, 19.2, 9.3, 8.9.
IR (ATR) $\boldsymbol{v}_{\mathbf{max}}$ = 2994.9, 2946.5, 2909.2, 1755.6, 1092.1, 1067.9, 1043.7, 1017.6, 997.1, 915.1, 810.7 cm^{-1}.
HPLC (CHIRALPAK IG-3 SFC, CO_2/MeOH = 95/5, flow rate = 2.0 mL/min, detector wavelength = 210 nm) t_R = 2.7 min (100%), 3.0 min (0%)
Specific rotation: $[\alpha]_D^{20}$ = +57.13 (deg·mL·g^{-1}·dm^{-1}) (measured in MeOH (10mg/mL) at 20 °C under 589 nm)

HRMS (ESI) ***m/z*:** calcd for $C_8H_{12}O_3 \cdot Na^+$ = $[M+Na]^+$ 179.0684, found 179.0680.

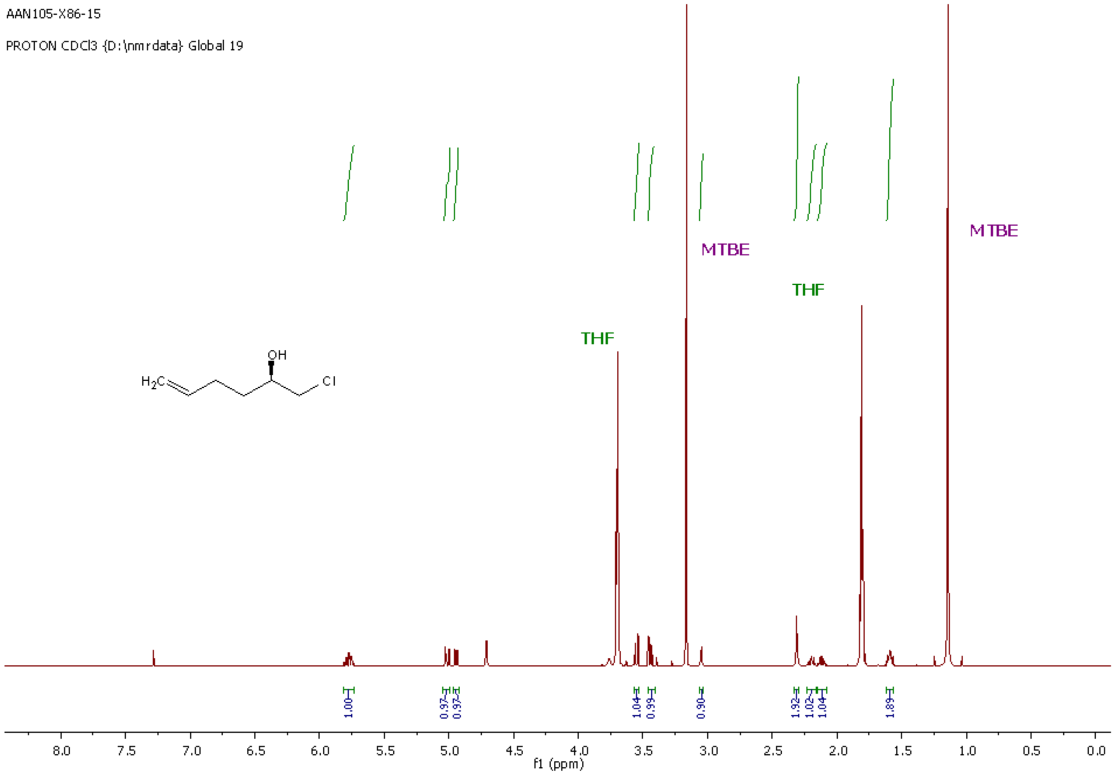

Figure S3. ^{1}HNMR (600MHz, $CDCl_3$) of **11**

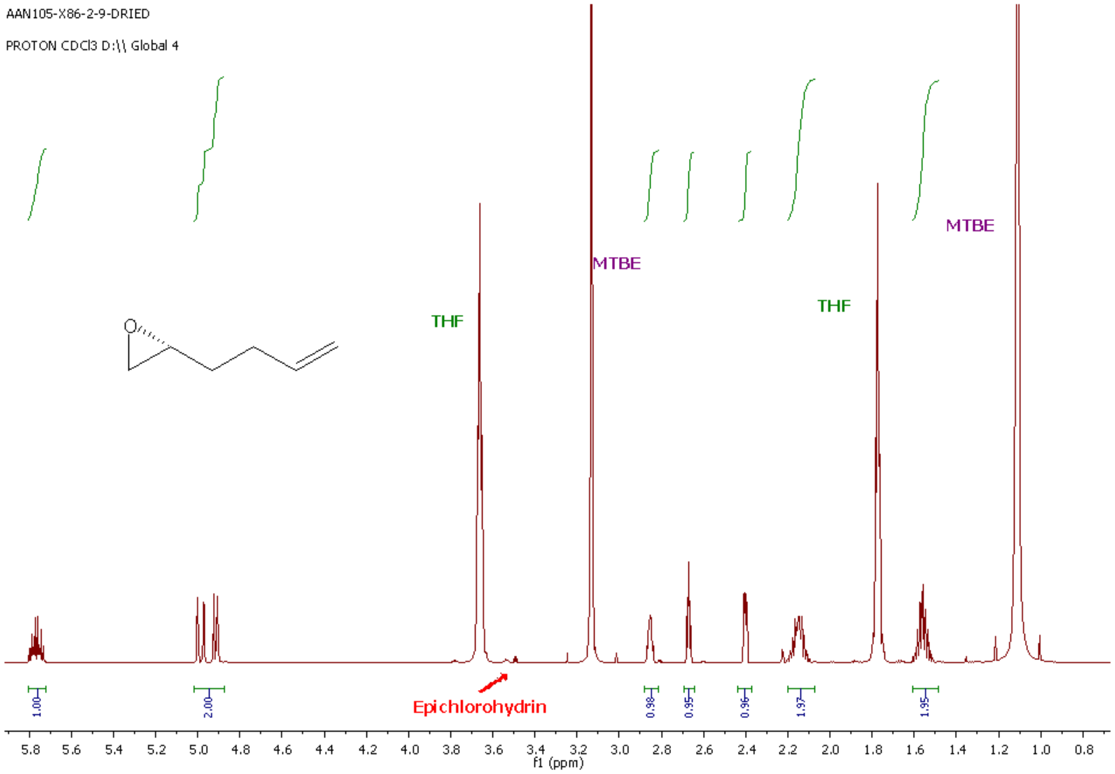

Figure S4. ^{1}HNMR (600MHz, $CDCl_3$) of **12**

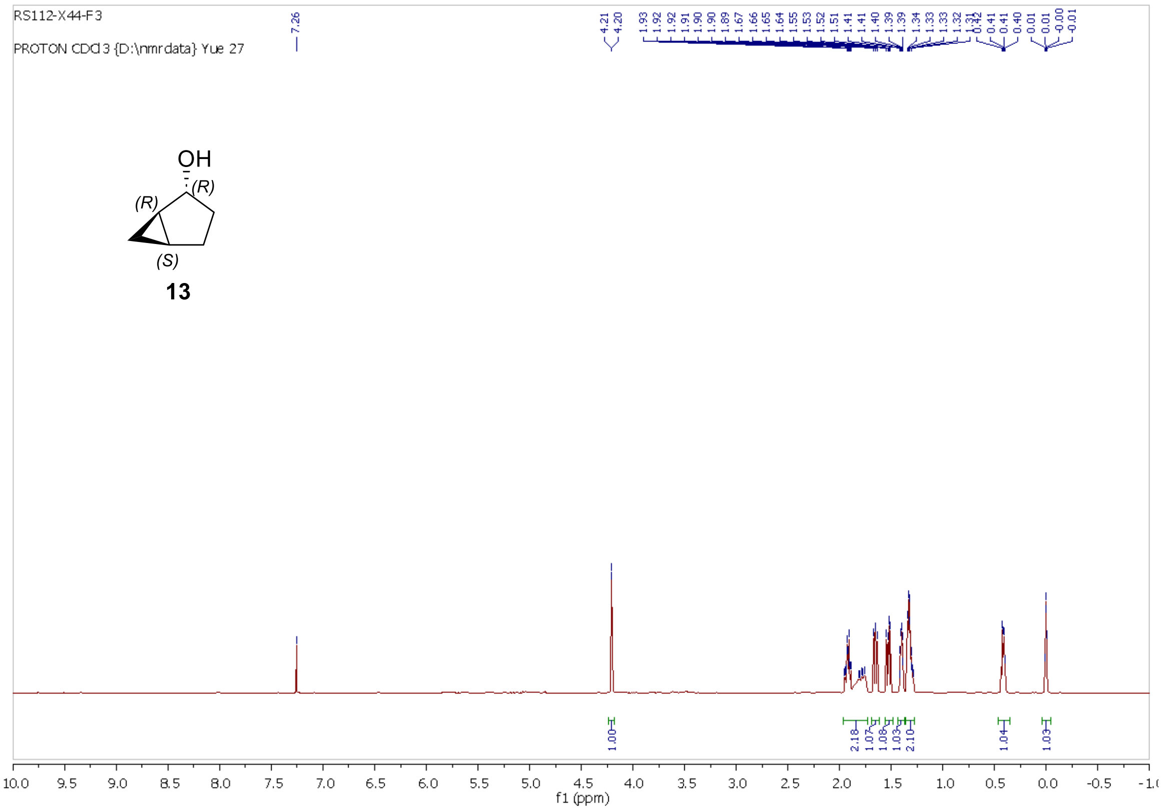

Figure S5. ^{1}HNMR (600MHz, $CDCl_3$) of **13**

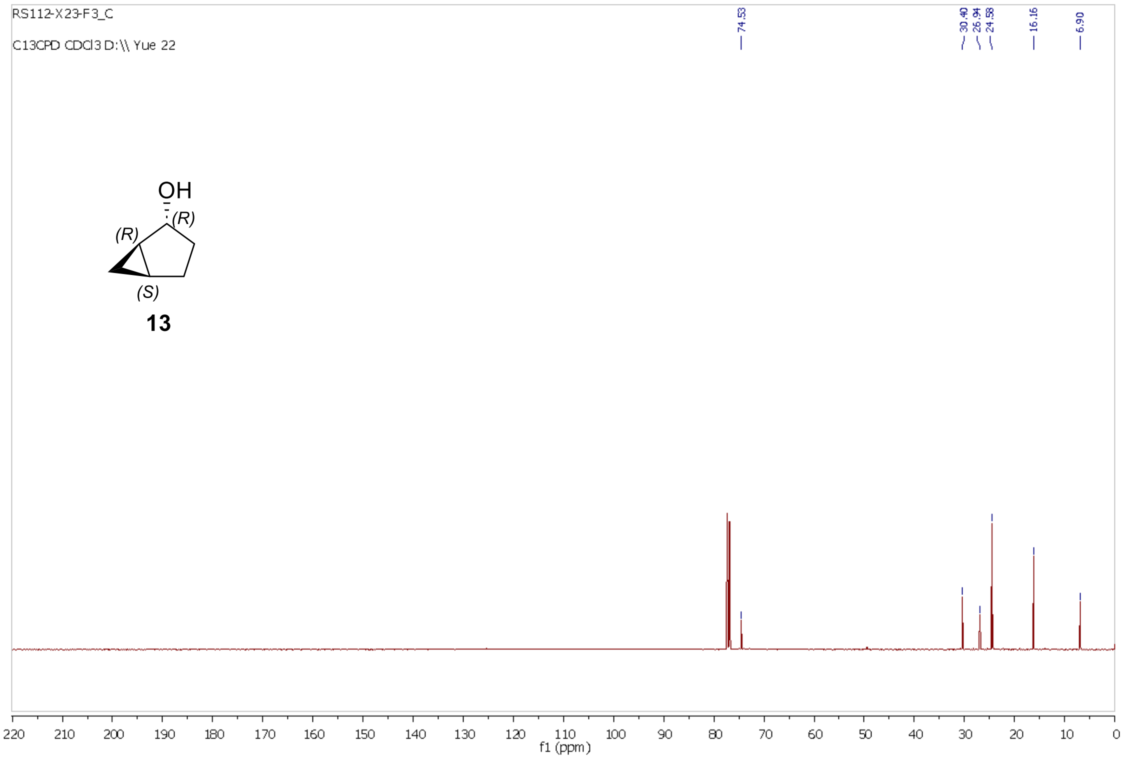

Figure S6. $^{13}C\{^{1}H\}$ NMR (150MHz, $CDCl_3$) of **13**

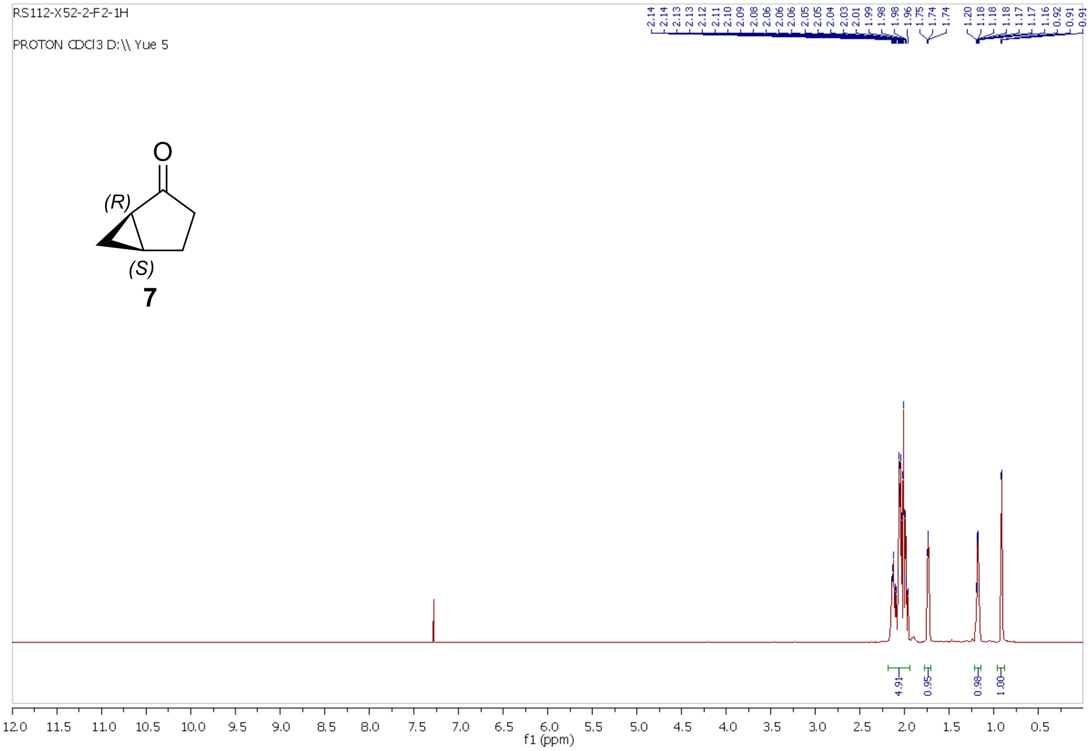

Figure S7. ^{1}HNMR (600MHz, $CDCl_3$) of **7**

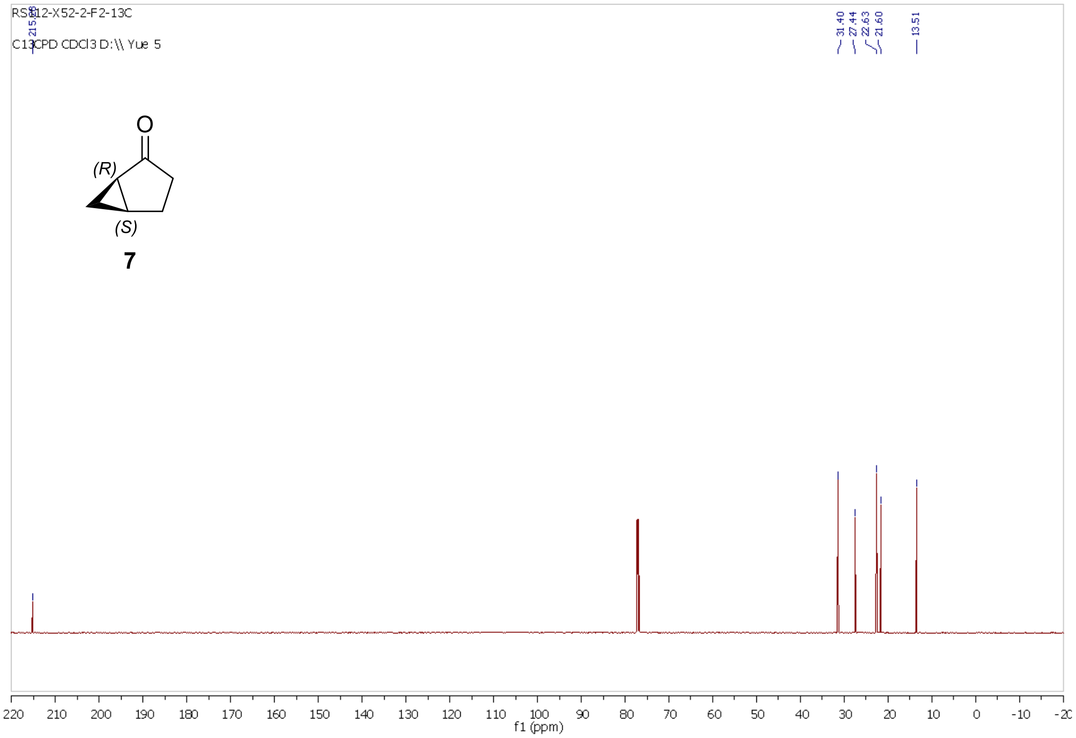

Figure S8. $^{13}C\{^1H\}$ NMR (150MHz, $CDCl_3$) of **7**

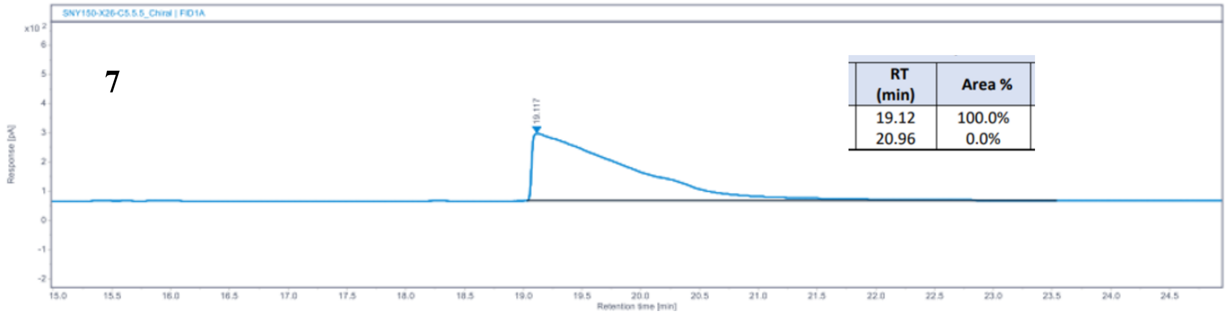

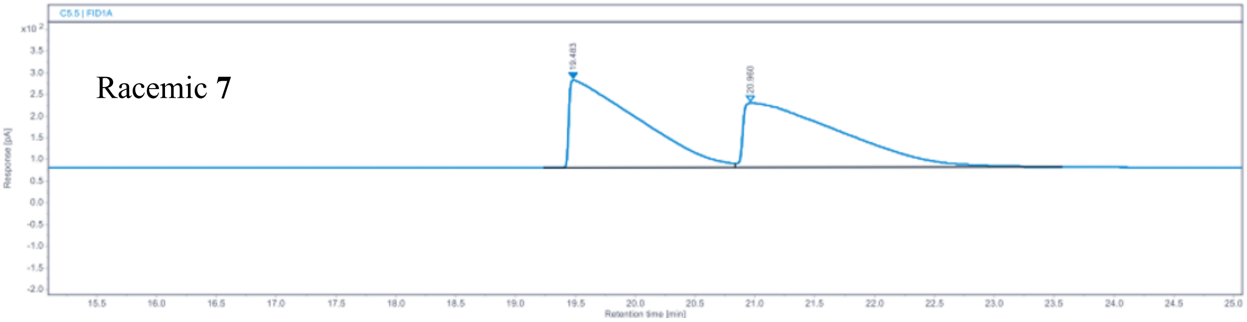

Figure S9**.** SFC spectra of compound **7**

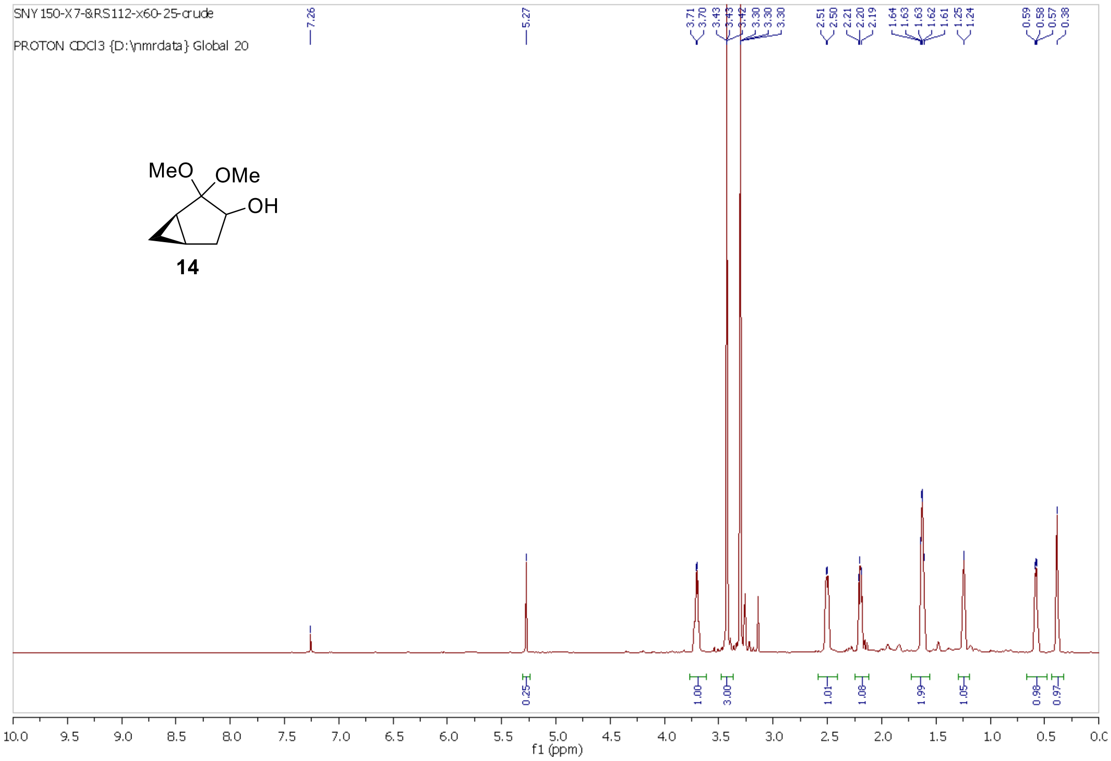

Figure S10. ^{1}HNMR (600MHz, $CDCl_3$) of **14**

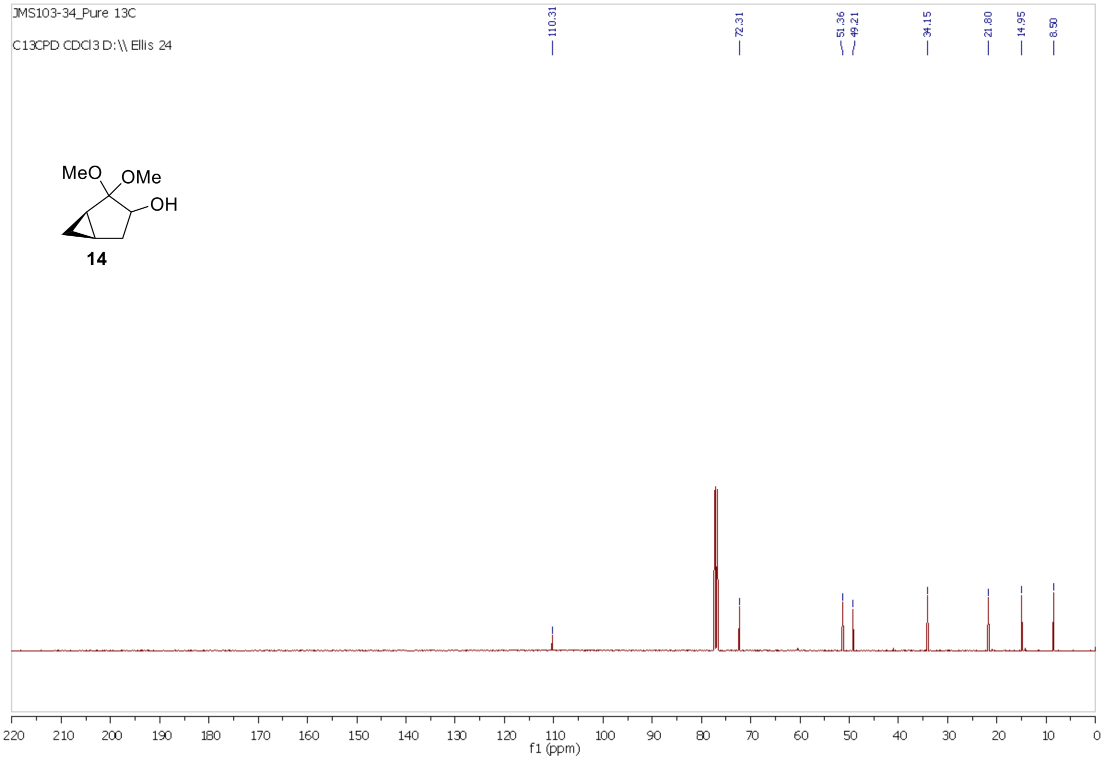

Figure S11. $^{13}C\{^{1}H\}$ NMR (150MHz, $CDCl_3$) of **14**

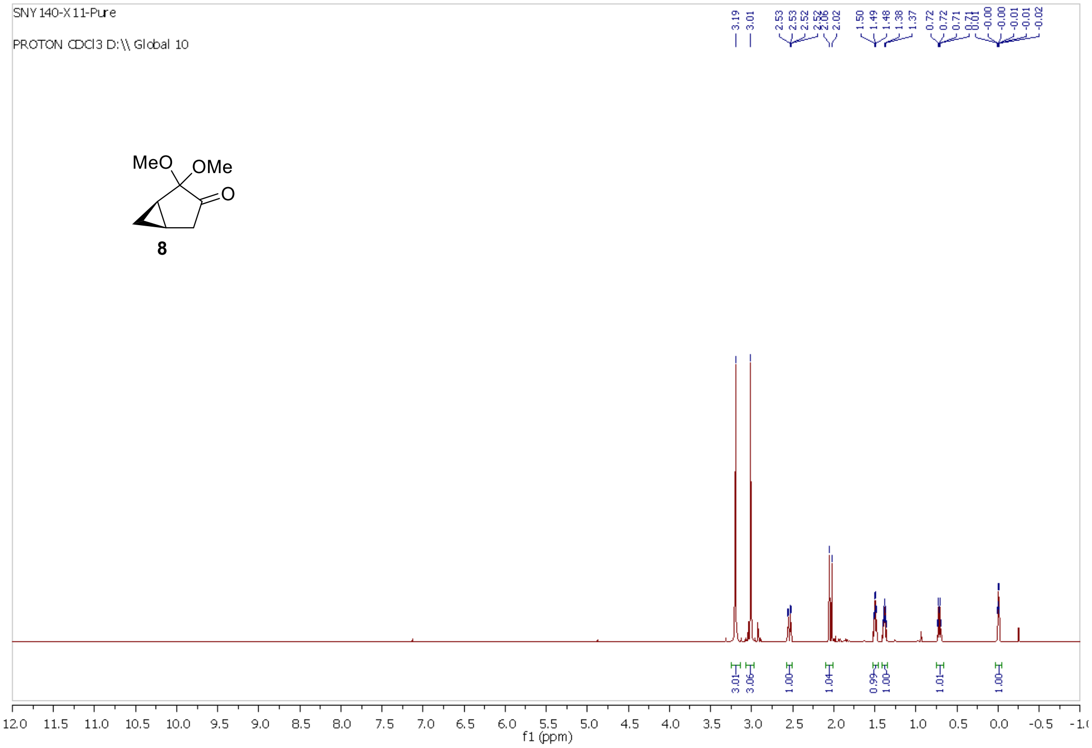

Figure S12. ^{1}HNMR (600MHz, $CDCl_3$) of **8**

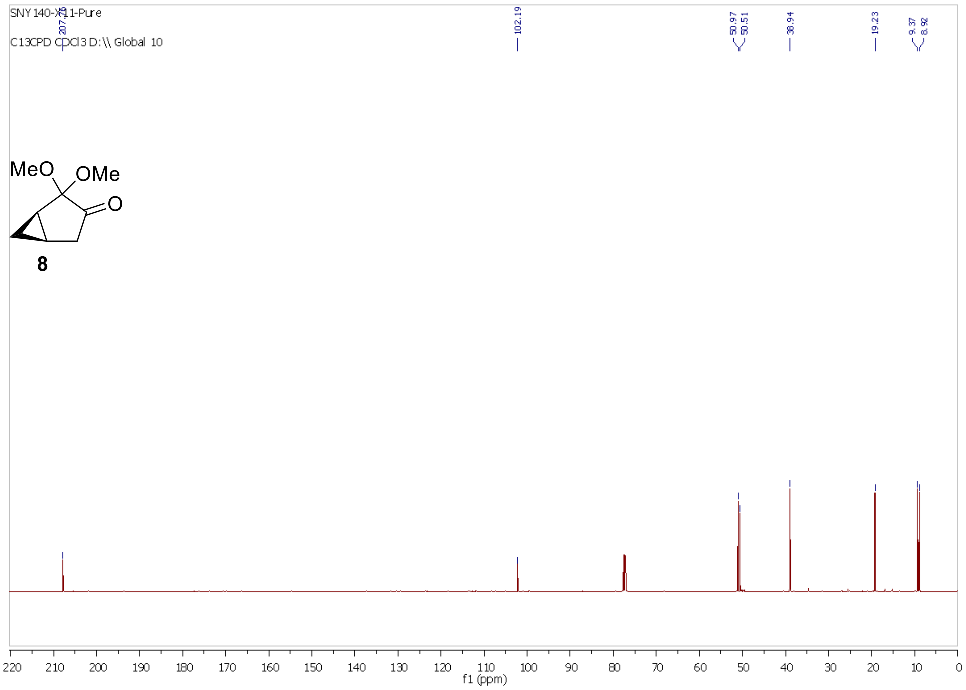

Figure S13. $^{13}C\{^1H\}$ NMR (150MHz, $CDCl_3$) of **8**

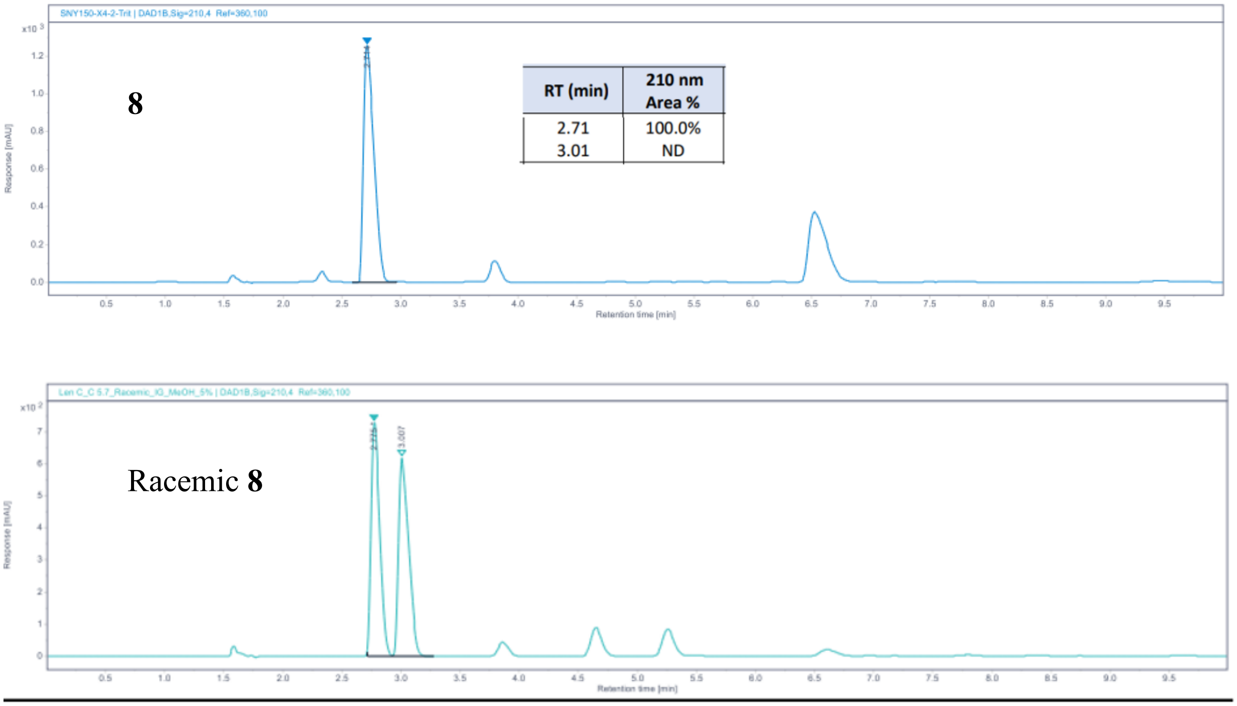

Figure S14. SFC spectra of compound **8**
